# Associations of the *miRNA-146a* rs2910164 and the *miRNA-499a* rs3746444 Polymorphisms With Plasma Lipid Levels: A Meta-Analysis

**DOI:** 10.3389/fgene.2021.746686

**Published:** 2021-09-27

**Authors:** Fuqiang Liu, Shengping Wang, Zhi Luo

**Affiliations:** ^1^Department of Cardiology, First People's Hospital of Chengdu, Chengdu, China; ^2^Department of Internal Medicine, Zhongnan Hospital of Wuhan University, Wuhan, China

**Keywords:** miRNA-146a, rs2910164, miRNA-499a, rs3746444, dyslipidemia

## Abstract

**Background:** The studies of miRNAs are vibrant and remain at the forefront in the cardiovascular system. Emerging studies indicate that the genetic polymorphisms of the *miRNA* gene may affect lipid metabolism; this study aims to clarify the specific correlations between the rs2910164 and rs3746444 polymorphisms and lipid levels.

**Methods and Results:** A comprehensive search of literature was performed from December 31, 2020, to May 31, 2021, by searching of the PubMed and the Cochrane databases. The standardized mean difference (SMD) and 95% confidence interval (CI) were used to evaluate the differences in lipid levels between the genotypes. rs2910164, a functional polymorphism in the *miRNA-146a* gene, was associated with increased triglycerides (TG) (SMD = 0.35, 95% CI = 0.15–0.54, *p* < 0.001), total cholesterol (TC) (SMD = 0.43, 95% CI = 0.16–0.70, *p* < 0.001), and low-density lipoprotein cholesterol (LDL-C) (SMD = 0.37, 95% CI = 0.11–0.63, *p* = 0.01) as well as decreased high-density lipoprotein cholesterol (HDL-C) (SMD = −0.27, 95% CI = −0.47−0.07, *p* = 0.01) levels. rs3746444, a functional polymorphism in the *miRNA-499a* gene, was only correlated with decreased TG (SMD = −0.09, 95% CI = −0.17−0.01, *P* = 0.03) levels.

**Conclusions:** The *miRNA-146a* rs2910164 polymorphism is significantly associated with atherogenic dyslipidemia.

## Introduction

MiRNAs play a central role in the posttranscriptional regulation of mRNA expression by binding to the 3′ untranslated region of mRNA, causing its destabilization, translational repression, or degradation.

Currently, a series of basic studies indicated that *miRNA-146a* and *miRNA-499a* may affect lipid metabolism. For instance, *miRNA-146a* knockout decreased the LDL-C levels (Cheng et al., 2017), while *miRNA-146a* overexpression induced severe dyslipidemia (Zhang et al., 2019). Moreover, macrophages transfected with *miRNA-146a* mimics reduced the intracellular LDL-C and oxLDL accumulation (Yang et al., 2011). However, transfection of HepG2 cells with the *miRNA-499a* inhibitor significantly increased the intracellular HDL-C levels (Chen et al., 2017). Together, it indicated that the expression levels of *miRNA-146a* and *miRNA-499a* may be closely linked to lipid metabolism.

The rs2910164 polymorphism is a function variant of *miRNA-146a*, formed by a nucleotide substitution from guanine (G) to cytosine (C). Studies showed that the C allele of the rs2910164 polymorphism largely increased the miRNA-146a protein levels (Shen et al., 2008; Ramkaran et al., 2014; Xiong et al., 2014). The rs3746444 polymorphism is a function variant of *miRNA-499a*, formed by a nucleotide substitution from adenine (A) to guanine (G). Evidence showed that the G allele of the rs3746444 polymorphism largely decreased the miRNA-499a protein levels (Chen et al., 2017). Therefore, the rs2910164 and rs3746444 polymorphisms may affect lipid levels by altering the expression levels of *miRNA-146a* and *miRNA-499a*. A series of meta-analyses (Labbaf et al., 2017; Liu et al., 2017; Zhou et al., 2017) showed that the subjects with rs2910164 and rs3746444 polymorphisms significantly increased the susceptibility to coronary artery disease (CAD). However, the underlying mechanisms remain elusive. Therefore, this study was required to clarify the mechanisms underlying the positive correlations between the rs2910164 and rs3746444 polymorphisms and CAD.

## Materials and Methods

The present meta-analysis is in accordance with the Preferred Reporting Items for Systematic Reviews and Meta-analyses (PRISMA) (Liberati et al., 2009).

### Literature Search

A comprehensive search of literature was performed from December 31, 2020, to May 31, 2021, by using seven databases including PubMed, Medline, Embase, Cochrane Library, Google Scholar, Foreign Medical Journal Service, and Excerpta Medica. The following keywords were used in the search: (“microRNA”, “miRNA”, “microRNA-146a”, “miRNA-146a”, “miR-146a”, “rs2910164”, “microRNA-499a”, “miRNA-499a”, “miR-499a”, or “rs3746444”), (“polymorphism”, “mutation”, “variation”, “mutant”, “variant”, “SNP”, or “single nucleotide polymorphism”), and (“lipids”, “circulating lipids”, “blood lipids”, “plasma lipids”, “serum lipids”, “triglycerides”, “total cholesterol”, “low-density lipoprotein cholesterol”, “high-density lipoprotein cholesterol”, “TG”, “TC”, “LDL-C”, or “HDL-C”). Additionally, the reference lists of all eligible studies were manually retrieved to obtain more literature.

### Inclusion Criteria

The specific inclusion criteria were listed as follows. (1) The articles investigated the effects of *miRNA-146a* rs2910164 (G > C) polymorphism or *miRNA-499a* rs3746444 (A > G) polymorphism on lipid levels. (2) The articles at least provided one parameter in the lipid profile (TG, TC, LDL-C, and HDL-C). (3) The articles provided the genotype frequencies of rs2910164 and rs3746444 polymorphisms. (4) The articles offered the mean lipid levels with standard deviation (SD) or standard errors (SE) by genotypes. (5) The interventional articles provided pre-intervention data. (6) The language of eligible articles was restricted to English or Chinese.

### Data Extraction

Two authors (SW and FL) extracted the data independently by using a standardized data extraction table. The discrepancy in data extracted was resolved by consensus or a discussion with the third author (ZL). If key data were absent, e-mail or telephone was used to contact the corresponding author to acquire these information.

One study conducted by Qiu et al. (2020) did not offer usable data in its original article; fortunately, they offered raw lipid data by the genotypes of rs2910164 and rs3746444 polymorphisms in its Supplementary Material. Therefore, we analysis and obtained these important data by performing SPSS software (version 23.0, Inc, Chicago, IL, USA).

The following data were extracted from each eligible article: the last name of the first author, year, country, gender, study population, ethnicity, genotype counts, genotyping methods, type of study, type of disease, total sample size, and mean lipid levels with SD or SE by genotypes.

### Data Analysis

The units of TG, TC, LDL-C, and HDL-C were converted into mmol/L. All extracted data were expressed as mean ± SD. The Hardy–Weinberg equilibrium (HWE) of the populations was tested by χ^2^ test. Since most of the included studies presented data in a dominant model [(GC + CC) vs. GG for rs2910164; (AG + GG) vs. AA for rs3746444], a dominant model was adopted to ensure adequate statistical power. All the analyses were performed by STATA software (version 15.0, College Station, TX, USA). *p* < 0.05 was recognized as statistically significant. The standardized mean difference (SMD) and 95% confidence interval (CI) were used to evaluate the differences in lipid levels between the genotypes. The raw data downloaded from Qiu et al. (2020) Supplementary Material were analyzed using SPSS software (version 23.0, Inc, Chicago, IL, USA). If data follow normal distribution, ANOVA was adopted to calculate the results.

### Heterogeneity Definition and Processing

Inevitably, there were differences between the included articles in a meta-analysis. The differences among participants, interventions, or inner authenticity variations in those articles were defined as “heterogeneity” (Higgins and Green, 2006). Heterogeneity was tested by I^2^ statistic and Cochran's χ2-based Q statistic. Galbraith plots were used to detect the potential sources of heterogeneity. If heterogeneity was significant (I^2^ > 50%, *p* ≤ 0.05), the random-effect model (DerSimonian–Laird method) was used to calculate the results (DerSimonian and Kacker, 2007). Otherwise, the fixed-effect model (Mantel–Haenszel method) would be adopted (I^2^ <50%, *p* > 0.05).

### Risk-of-Bias Test

The risk of bias among the included studies was evaluated by the risk-of-bias plot (Savović et al., 2014), in which different colors represent different levels of risk of bias, e.g., green represents low risk of bias, while yellow refers to unclear risk of bias; however, red represents high risk of bias.

### Publication Bias Test

The publication bias among the included studies was evaluated by Begg's funnel plot and Egger's linear regression test (Begg and Mazumdar, 1994). The funnel plots were asymmetric when there were publication biases and symmetric in case of no publication bias.

### Subgroup Analysis

Subgroup analysis was carried out by ethnicity, female subjects, and cardiovascular disease (CVD) patients due to the limited number of studies. The ethnicity was divided into Caucasian and Asian. The subjects with CAD, ischemic stroke (IS), or hypertension were considered as CVD patients. In some studies, the subjects were divided into more than one subpopulation (e.g., the subjects with different types of disease, the subjects originated from different races, case and control subjects). Each subpopulation was regarded as an independent comparison in this study.

### Sensitivity Analysis

Sensitivity analysis was conducted in this meta-analysis, in which the comparison was excluded one by one, and the analysis was performed again after omitting each comparison. If the synthetic results in any of the comparison changed substantially to alter the results from significant to non-significant or the other way around. The absence of such a phenomenon usually indicates the robustness and stableness of synthetic results.

## Results

### Study Selection

**Figure 1:**
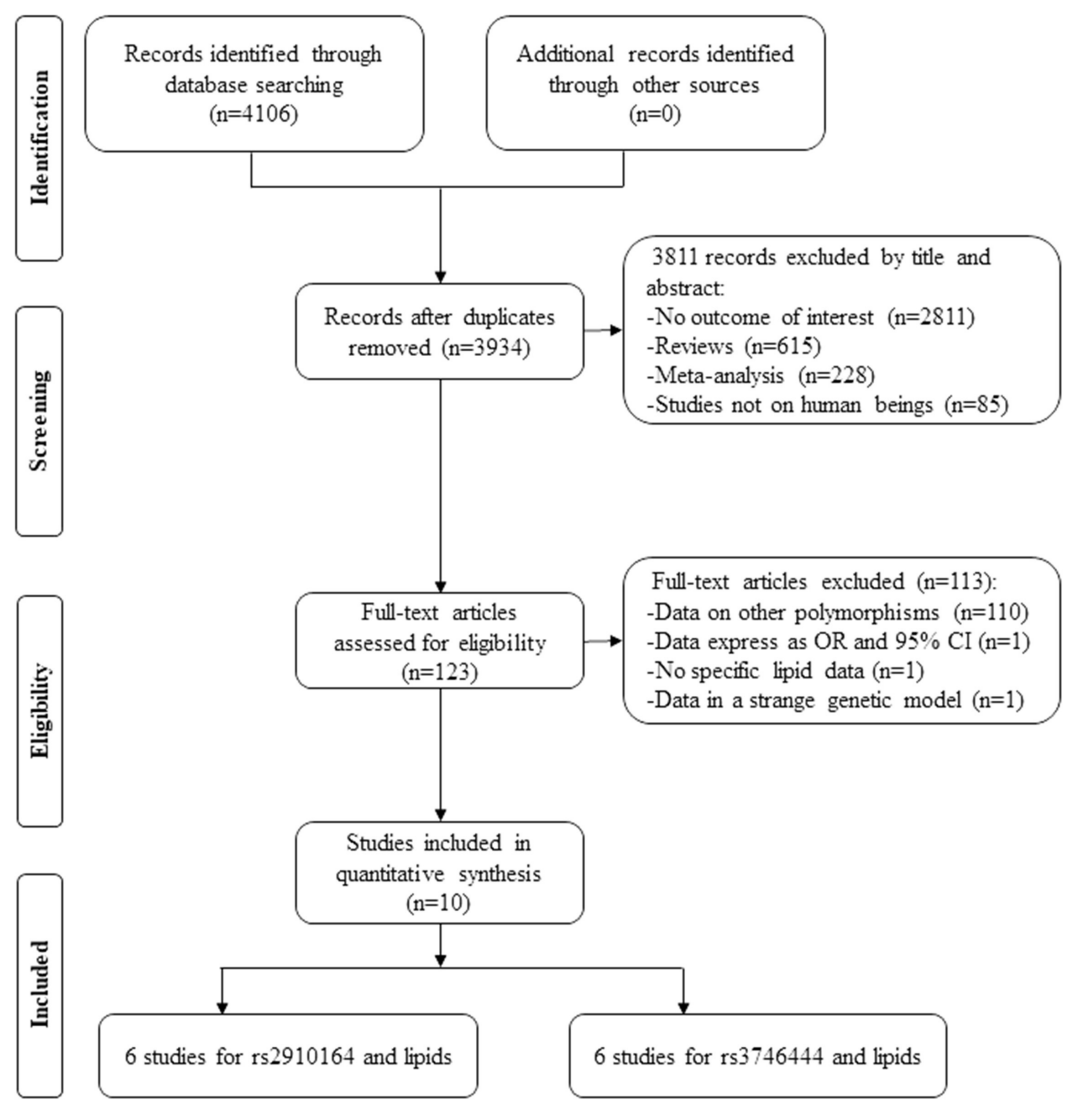
Flow diagram of the articles selection process.

By searching relevant databases, 4,106 articles were identified; after the screening, 3,811 articles were excluded by its title and abstract. Next, 123 articles were further estimated by its contents, in which 110 articles provided data of other polymorphisms, one article conducted by Mir et al. (2020) did not provide the specific lipid levels by the genotypes of rs2910164, one article conducted by Huang et al. (2015) provided lipid levels by the genotypes of rs2910164 but expressed odds ratio (OR) and 95% CI, and 1 article conducted by Ibrahim et al. (2020) provided lipid levels by the genotypes of rs2910164 but in a strange genetic model [(CG + GG) vs. CC]. Therefore, 113 articles were further excluded. Despite that we carefully and repeatedly reviewed all the relevant literatures and tried our best to obtain more studies, only 10 studies (4,087 subjects) were included due to the limited number of eligible studies, in which, six studies (3,470 subjects) and six studies (3,227 subjects) were respectively included for lipid association analysis in rs2910164 and rs3746444 (Figure 1).

The references of the included studies are listed in Supplementary Material. The characteristics of the studies included in the meta-analysis are presented in Supplementary Table S1. The plasma lipid levels by the genotypes of *miRNA-146a* rs2910164 polymorphism are presented in Supplementary Table S2. The plasma lipid levels by the genotypes of *miRNA-499a* rs3746444 polymorphism are presented in Supplementary Table S3. The forest plot of the meta-analysis between *miRNA-499a* rs3746444 polymorphism and plasma lipid levels is presented in Supplementary Figures S1–S4. The sensitivity analysis between *miRNA-499a* rs3746444 polymorphism and plasma lipid levels is presented in Supplementary Figures S5–S7, S11. The sensitivity analysis between *miRNA-146a* rs2910164 polymorphism and plasma lipid levels is presented in Supplementary Figures S8–S10, S12. The risk-of-bias plot of the meta-analysis between *miRNA-499a* rs3746444 polymorphism and plasma lipid levels is presented in Supplementary Figure S13. The Begg's funnel plot of the association analysis between *miRNA-146a* rs2910164 polymorphism and plasma TC, LDL-C, and HDL-C levels is presented in Supplementary Figures S14–S16, respectively. The Begg's funnel plot of the association analysis between *miRNA-499a* rs3746444 polymorphism and plasma lipid levels is presented in Supplementary Figures S17–S20.

### Associations of the rs2910164 Polymorphism With Lipid Levels

**Figure 2:**
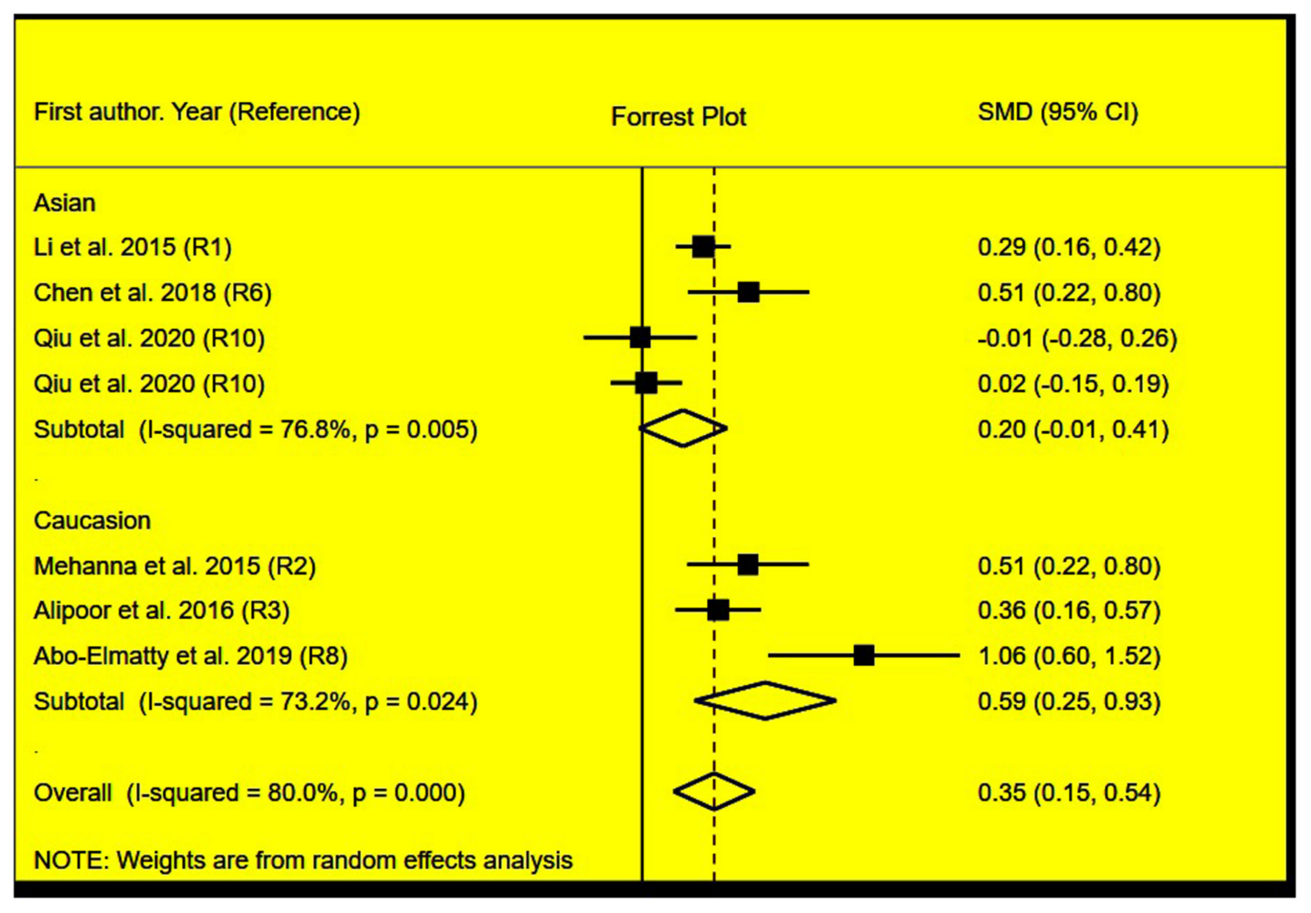
Forest plot of the meta-analysis between *miRNA-146a* rs2910164 polymorphism and plasma TG levels.

**Figure 3:**
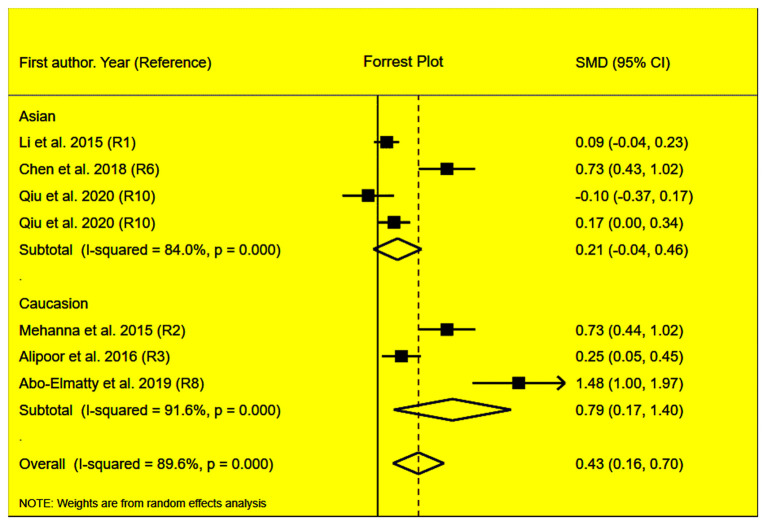
Forest plot of the meta-analysis between *miRNA-146a* rs2910164 polymorphism and plasma TC levels.

**Figure 4:**
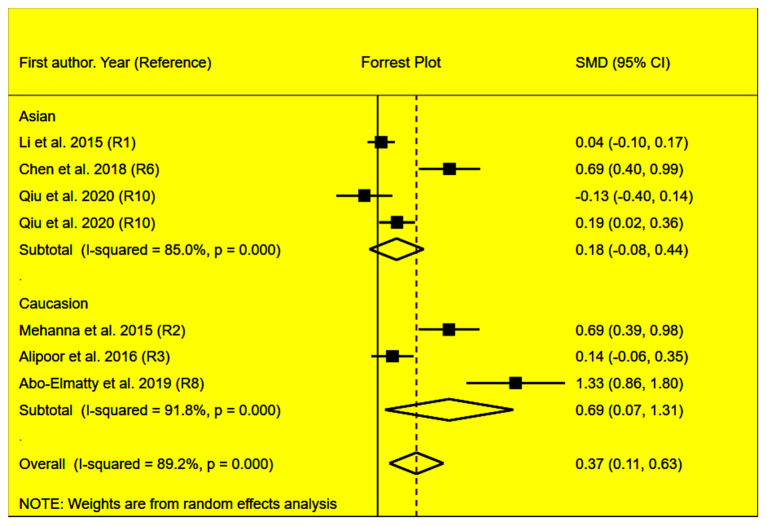
Forest plot of the meta-analysis between *miRNA-146a* rs2910164 polymorphism and plasma LDL-C levels.

**Figure 5:**
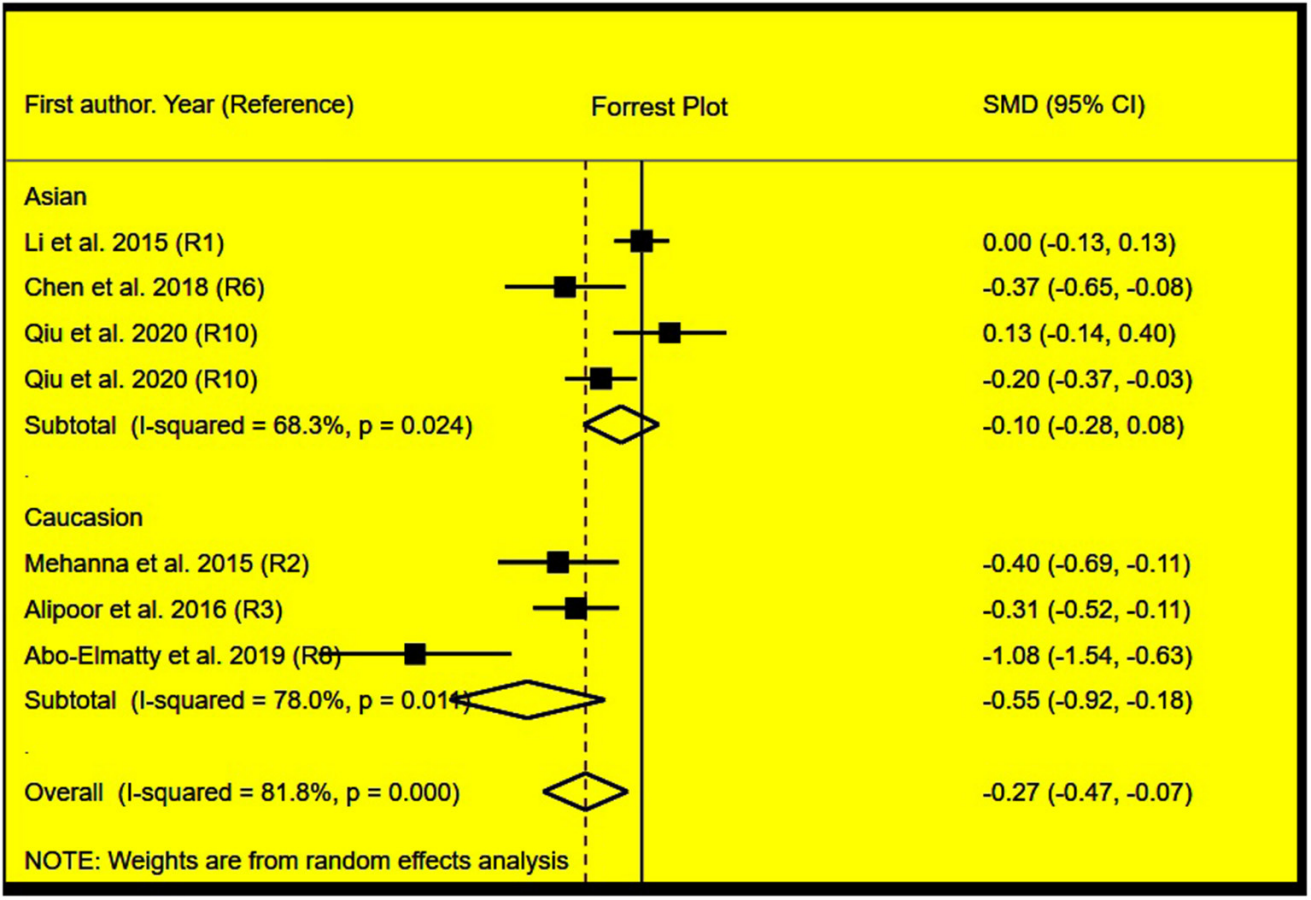
Forest plot of the meta-analysis between *miRNA-146a* rs2910164 polymorphism and plasma HDL-C levels.

The rs2910164 polymorphism largely increased the TG (SMD = 0.35, 95% CI = 0.15–0.54, *p* < 0.001), TC (SMD = 0.43, 95% CI = 0.16–0.70, *p* < 0.001), and LDL-C (SMD = 0.37, 95% CI = 0.11–0.63, *p* = 0.01) levels and decreased the HDL-C level (SMD = –0.27, 95% CI = −0.47−0.07, *p* = 0.01) (Table 1; Figures 2–5). When the analysis was limited to the studies in HWE, the significant associations of the rs2910164 polymorphism with TG, TC, LDL-C, and HDL-C levels were also detected (Table 1).

**Table 1:** Meta-analysis of the *miRNA-146a* rs2910164 polymorphism with lipid levels.

**Groups or subgroups**	**SMD**	**95% CI**	***p*-value**
**TG**			
All	0.35	0.15–0.54	<0.001
Studies in HWE	0.35	0.12–0.58	<0.01
Caucasian	0.59	0.25–0.93	<0.01
Asian	0.20	−0.01–0.41	0.07
Females	0.76	0.21–1.30	0.01
CVD	0.40	−0.03–0.83	0.06
**TC**			
All	0.43	0.16–0.70	<0.001
Studies in HWE	0.47	0.15–0.80	<0.01
Caucasian	0.79	0.17–1.40	0.01
Asian	0.21	−0.04–0.46	0.10
Females	1.08	0.35–1.82	<0.01
CVD	0.44	−0.18–1.07	0.16
**LDL-C**			
All	0.37	0.11–0.63	0.01
Studies in HWE	0.42	0.11–0.74	0.01
Caucasian	0.69	0.07–1.31	0.03
Asian	0.18	−0.08–0.44	0.17
Females	0.98	0.35–1.61	<0.01
CVD	0.36	−0.22–0.95	0.22
**HDL-C**			
All	−0.27	−0.47 −0.07	0.01
Studies in HWE	−0.27	−0.50−0.03	0.03
Caucasian	−0.55	−0.92−0.18	<0.01
Asian	−0.10	−0.27–0.08	0.28
Females	−0.72	1.39−0.05	0.03
CVD	−0.27	−0.77–0.24	0.30

*miRNA, microRNA; SMD, standardized mean difference; 95% CI, 95% confidence interval; HWE, hardy–weinberg equilibrium; TG, triglyceride; TC, total cholesterol; LDL-C, low-density lipoprotein cholesterol; HDL-C, high-density lipoprotein cholesterol; CVD, cardiovascular disease*.

Then, the subgroup analysis by the characteristics of the subjects (Table 1) showed that the significant associations of the rs2910164 polymorphism with TG, TC, LDL-C, and HDL-C levels were only observed in Caucasians and females (Table 1). In addition, a marginal significance was observed in patients with CVD in the association analysis between the rs2910164 polymorphism and TG levels (Table 1).

The analysis that excluded the studies with heterogeneity was also carried out (Table 2), and the analysis results showed that a significant association of the rs2910164 polymorphism with TG levels was observed in Caucasians, Asians, and CVD patients, while a significant association of the rs2910164 polymorphism with TC levels was only observed in Asians. Moreover, a significant association of the rs2910164 polymorphism with HDL-C levels was observed in Caucasians and Asians. However, only a marginal significance was observed in the analysis of the correlation between the rs2910164 polymorphism and LDL-C levels.

**Table 2:** Meta-analysis of the *miRNA-146a* rs2910164 polymorphism with lipid levels (after excluding the study with heterogeneity).

**Groups or subgroups**	**SMD**	**95% CI**	***p*-value**
**TG**			
All	0.32	0.22–0.41	<0.001
Studies in HWE	0.30	0.20–0.41	<0.001
Caucasian	0.41	0.24–0.58	<0.001
Asian	0.28	0.17–0.38	<0.001
Females	–	–	–
CAD	0.24	0.12–0.35	<0.001
**TC**			
All	0.12	0.04–0.21	0.01
Studies in HWE	0.10	0.00–0.19	0.05
Caucasian		–	–
Asian	0.10	0.00–0.19	0.05
Females	–	–	–
CVD	0.06	−0.06–0.18	0.33
**LDL-C**			
All	0.08	−0.01–0.17	0.08
Studies in HWE	0.06	−0.03–0.16	0.20
Caucasian	–	–	–
Asian	0.06	−0.03–0.16	0.20
Females	–	–	–
CVD	0.00	−0.11–0.12	0.94
**HDL-C**			
All	−0.29	−0.40−0.18	<0.001
Studies in HWE	−0.27	−0.40−0.14	<0.001
Caucasian	−0.34	−0.51−0.18	<0.001
Asian	−0.24	−0.39−0.09	<0.01
Females	–	–	–
CVD	–	–	–

*miRNA, microRNA; SMD, standardized mean difference; 95% CI, 95% confidence interval; HWE, hardy–weinberg equilibrium; TG, triglyceride; TC, total cholesterol; LDL-C, low-density lipoprotein cholesterol; HDL-C, high-density lipoprotein cholesterol; CAD, coronary artery disease; CVD, cardiovascular disease*.

### Associations of the rs3746444 Polymorphism With Lipid Levels

No statistical significance was observed in the analysis of associations of the rs3746444 polymorphism with TG (Supplementary Figure S1), TC (Supplementary Figure S2), LDL-C (Supplementary Figure S3), and HDL-C (Supplementary Figure S4) levels (Table 3). However, after excluding the studies with heterogeneity, the correlation between the rs3746444 polymorphism and TG levels showed statistical significance in Asians and CVD patients (see Table 4 for more details).

**Table 3:** Meta-analysis of the *miRNA-499a* rs3746444 polymorphism with lipid levels.

**Groups or subgroups**	**SMD**	**95% CI**	***p*-value**
**TG**			
All	−0.02	−0.15–0.11	0.75
Studies in HWE	−0.02	−0.13–0.08	0.68
Asian	−0.03	−0.17–0.11	0.66
CVD	−0.02	−0.22–0.18	0.83
**TC**			
All	−0.03	−0.10–0.05	0.46
Studies in HWE	−0.01	−0.12–0.09	0.83
Asian	−0.02	−0.10–0.06	0.59
CVD	−0.03	−0.13–0.06	0.51
**LDL-C**			
All	0.02	−0.06–0.10	0.61
Studies in HWE	0.01	−0.10–0.12	0.88
Asian	0.02	−0.06–0.10	0.61
CVD	0.03	−0.07–0.13	0.54
**HDL-C**			
All	0.10	−0.13–0.32	0.40
Studies in HWE	0.24	−0.16–0.64	0.23
Asian	0.15	−0.09–0.40	0.23
CVD	−0.01	−0.13–0.11	0.86

*miRNA, microRNA; SMD, standardized mean difference; 95% CI, 95% confidence interval; HWE, hardy–weinberg equilibrium; TG, triglyceride; TC, total cholesterol; LDL-C, low-density lipoprotein cholesterol; HDL-C, high-density lipoprotein cholesterol; CVD, cardiovascular disease*.

**Table 4:** Meta-analysis of the *miRNA-499a* rs3746444 polymorphism with lipid levels (after excluding the study with heterogeneity).

**Groups or subgroups**	**SMD**	**95% CI**	***p*-value**
**TG**			
All	−0.09	−0.17−0.01	0.03
Studies in HWE	−0.02	−0.13–0.08	0.68
Asian	−0.28	−0.17−0.38	<0.001
CVD	−0.15	−0.26−0.04	0.01
**TC**			
All	−0.03	−0.10–0.05	0.46
Studies in HWE	−0.01	−0.12–0.09	0.83
Asian	−0.02	−0.10–0.06	0.59
CVD	−0.03	−0.13–0.06	0.51
**LDL-C**			
All	0.02	−0.06–0.10	0.61
Studies in HWE	0.01	−0.10–0.12	0.88
Asian	0.02	−0.06–0.10	0.61
CVD	0.03	−0.07–0.13	0.54
**HDL-C**			
All	−0.03	−0.11–0.04	0.38
Studies in HWE	−0.07	−0.18–0.03	0.17
Asian	−0.02	−0.10–0.06	0.57
CVD	−0.00	−0.09–0.09	0.97

*miRNA, microRNA; SMD, standardized mean difference; 95% CI, 95% confidence interval; HWE, hardy–weinberg equilibrium; TG, triglyceride; TC, total cholesterol; LDL-C, low-density lipoprotein cholesterol; HDL-C, high-density lipoprotein cholesterol, CVD, cardiovascular disease*.

### Evaluation of Heterogeneity

In the analysis of correlations between the rs2910164 polymorphism and lipid levels, significant heterogeneity was detected in TG, TC, LDL-C, and HDL-C (Table 1). Two (Abo-Elmatty and Mehanna, 2019; Qiu et al., 2020), three (Mehanna et al., 2015; Chen et al., 2018; Abo-Elmatty and Mehanna, 2019), three (Mehanna et al., 2015; Chen et al., 2018; Abo-Elmatty and Mehanna, 2019), and three (Li et al., 2015a; Abo-Elmatty and Mehanna, 2019; Qiu et al., 2020) comparisons were recognized as the main contributors to TG, TC, LDL-C, and HDL-C heterogeneity, respectively. SMD values and 95% CIs of TG, TC, and HDL-C did not change substantially after excluding these comparisons (Table 2). However, the SMD value and 95% CI of LDL-C (SMD = 0.08, 95% CI = −0.01–0.17, *p* = 0.08) changed significantly after excluding these outlier comparisons.

In the correlation analysis between the rs3746444 polymorphism and lipid levels, significant heterogeneity was detected in TG and HDL-C (Table 3). One comparison (Qiu et al., 2020) and one comparison (Chen et al., 2017) were recognized as the main contributors to TG and HDL-C heterogeneity, respectively. The SMD value and 95% CI of HDL-C did not change substantially after excluding this comparison (Chen et al., 2017). However, the SMD value and 95% CI of TG (SMD = −0.09, 95% CI = −0.17−0.01, *p* = 0.03) changed significantly after excluding this comparison (Qiu et al., 2020).

### Sensitivity Analysis

Sensitivity analysis showed that no comparison may affect the associations of the rs3746444 polymorphism with the TC (Supplementary Figure S5), LDL-C (Supplementary Figure S6), and HDL-C (Supplementary Figure S7) levels. However, one comparison (Li et al., 2015a) may affect the significant associations of the rs2910164 polymorphism with the TC (Supplementary Figure S8), LDL-C (Supplementary Figure S9), and HDL-C (Supplementary Figure S10) levels as well as the significant correlation between the rs3746444 polymorphism and TG (Supplementary Figure S11) levels, while another comparison (Qiu et al., 2020) may affect the correlation between the rs2910164 polymorphism and TG (Supplementary Figure S12) levels.

The correlations between the rs2910164 and rs3746444 polymorphisms and lipid levels did not change substantially [rs2910164 and TG levels: (SMD = 0.41, 95% CI = 0.21–0.61, *p* <0.001); rs2910164 and TC levels: (SMD = 0.50, 95% CI = 0.17–0.84, *p* < 0.01); rs2910164 and LDL-C levels: (SMD = 0.45, 95% CI = 0.13–0.77, *p* = 0.01); rs2910164 on HDL-C levels: (SMD = −0.33, 95% CI = −0.55−0.10, *p* < 0.01); rs3746444 and TG levels: (SMD = 0.03, 95% CI = −0.07–0.13, *p* = 0.60)] after omitting above comparisons (Li et al., 2015a; Qiu et al., 2020). It indicated that the present analysis results were robust and stable.

### Risk-of-Bias Test

**Figure 6:**
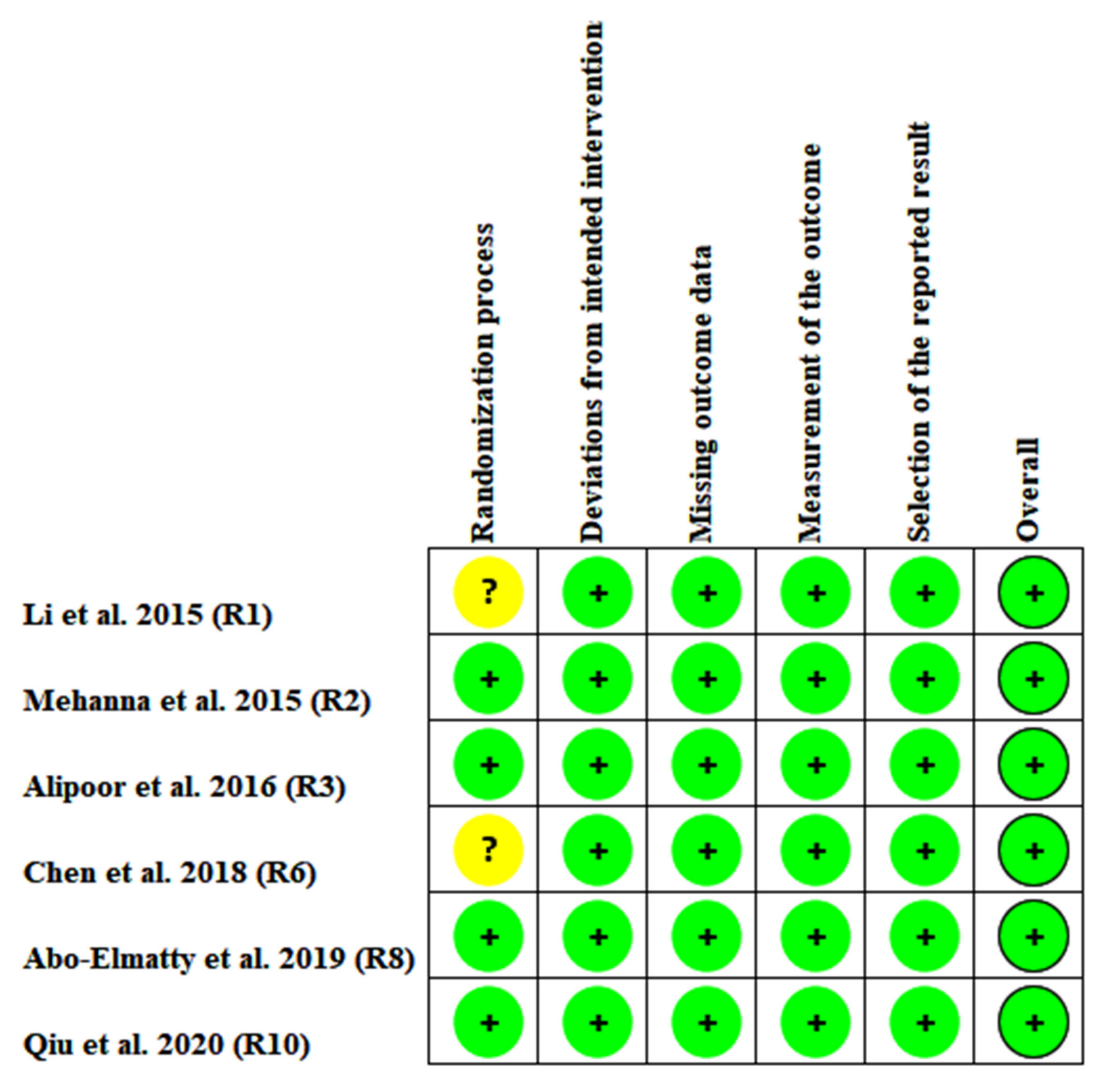
Risk bias plot of the meta-analysis between *miRNA-146a* rs2910164 polymorphism and plasma lipid levels (for assessment of each entry, green represents low risk of bias and yellow refers to unclear risk of bias).

In the association analysis between the rs3746444 polymorphism and lipid levels, some concerns were observed in the randomization process (33.3%) and measurement of the outcome (16.7%); however, the overall results showed a low risk of bias (66.7%) among the included studies (Supplementary Figure S13). It indicated that the studies included in the meta-analysis were of relatively high quality. In addition, there was no risk of bias in the analysis of association of the rs3746444 polymorphism with lipid levels in addition to some concerns (33.3%) in the randomization process (Figure 6).

### Publication Bias Test

**Figure 7:**
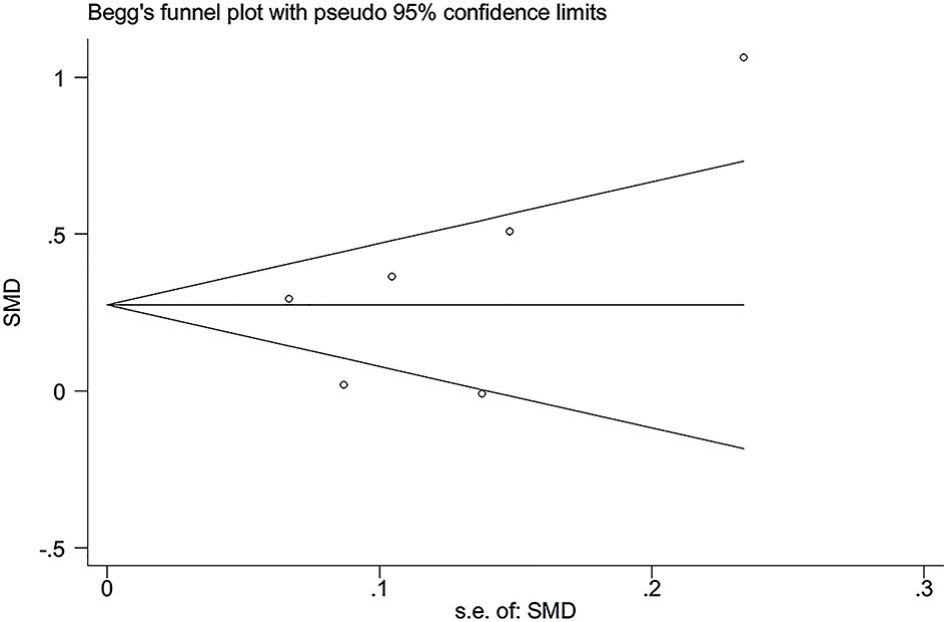
Begg's funnel plot of the association analysis between *miRNA-146a* rs2910164 polymorphism and plasma TG levels.

In the present study, all the funnel plots were symmetric visually (Figure 7; Supplementary Figures S14–S20). Begg's test did not find any publication bias in the analysis for the rs2910164 and rs3746444 polymorphisms, which was confirmed by Egger's regression test [*p* = 0.22 for rs2910164 and TG (Figure 7), *p* = 0.06 for rs2910164 and TC (Supplementary Figure S14), *p* = 0.06 for rs2910164 and LDL-C (Supplementary Figure S15), *p* = 0.07 for rs2910164 and HDL-C (Supplementary Figure S16), *p* = 0.34 for rs3746444 and TG (Supplementary Figure S17), *p* = 0.27 for rs3746444 and TC (Supplementary Figure S18), *p* = 0.10 for rs3746444 and LDL-C (Supplementary Figure S19), *p* = 0.18 for rs3746444 and HDL-C (Supplementary Figure S20)].

## Discussion

The present study showed that the rs2910164 polymorphism of *miRNA-146a* was significantly associated with increased TG, TC, and LDL-C levels as well as decreased HDL-C levels in Caucasians and females. However, the rs3746444 polymorphism of *miRNA-499a* only decreased the TG levels in Asians and CVD patients.

The underlying mechanisms whereby the rs2910164 polymorphism of *miRNA-146a* affects lipid levels largely depended on the following pathways: (1) by attenuating toll-like receptor 4 (TLR4) counts. TLR4 is a known target of *miRNA-146a* and plays a key role in lipid uptake (Choi et al., 2009); the increase of miRNA-146a protein caused by the rs2910164 polymorphism (Shen et al., 2008; Ramkaran et al., 2014; Xiong et al., 2014) inhibits the expression of TLR4 (Shen et al., 2008), thus reducing lipid uptake and resulting in dyslipidemia. (2) By attenuating the sortilin-1 (Sort1) expression. Sort1 is a novel target of *miRNA-146a* and contributes to LDL-C degradation (Musunuru et al., 2010); the increase of miRNA-146a expression caused by the rs2910164 polymorphism (Shen et al., 2008; Ramkaran et al., 2014; Xiong et al., 2014) may inhibit the expression of Sort1 (Cheng et al., 2017), thus repressing the degradation of LDL-C in plasma. (3) By attenuating the expression of interleukin-1 receptor-associated kinase-1 (IRAK-1) and dysfunction of ATP-binding cassette transporter A1 (ABCA1)/ATP-binding cassette transporter G1 (ABCG1). IRAK-1 is a regulator of ABCA1/ABCG1 (Maitra et al., 2009); the increase of miRNA-146a expression caused by the rs2910164 polymorphism (Shen et al., 2008; Ramkaran et al., 2014; Xiong et al., 2014) may induce the dysfunction of ABCA1/ABCG1 by downregulating the expression of IRAK-1 (Li et al., 2015b). It is well-known that ABCA1/ABCG1 plays a central role in reverse cholesterol transport (RCT); the dysfunction of ABCA1/ABCG1 caused by the rs2910164 polymorphism may therefore result in dyslipidemia. The mechanisms underlying the rs3746444 polymorphism which reduced the TG levels have not been clarified yet. However, osbpl1a, a target of *miRNA-499a* (Chen et al., 2017), has been proved to play a crucial role in lipid metabolism regulation. The largely decreased miRNA-499a protein levels caused by the rs3746444 polymorphism (Chen et al., 2017) may increase the expression levels of osbpl1a, thus decreasing the TG levels.

Atherogenic dyslipidemia is characterized by increased levels of TG, TC, and LDL-C and/or decreased level of HDL-C in plasma. In the present study, the rs2910164 polymorphism significantly increased the plasma levels of TG, TC, and LDL-C and significantly decreased the plasma levels of HDL-C (Table 1). It indicated that the rs2910164 polymorphism of the *miRNA-146a* gene was significantly associated with atherogenic dyslipidemia, considering that atherogenic dyslipidemia is one of the most important risk factors for CAD and accounts for at least 50% of the population-attributable risk (Yusuf et al., 2004). It is not difficult to speculate that the positive correlation between the rs2910164 polymorphism and CAD (Liu et al., 2017; Zhou et al., 2017) was mediated, at least partly, by atherogenic dyslipidemia. Interestingly, this speculation was verified in our study whereby the largely increased TG levels caused by the rs2910164 polymorphism were observed in CAD patients (Table 2). More importantly, whereas the correlation between the rs2910164 polymorphism and atherogenic dyslipidemia was robust and strong (Tables 1, 2), it indicated that the rs2910164 polymorphism of the *miRNA-146a* gene may be a new therapeutic target for CAD.

Regarding the rs3746444 polymorphism, only decreased TG levels were observed (Table 4); therefore, the positive correlation between this polymorphism and increased CAD risk (Labbaf et al., 2017) could not be interpreted by its effect on lipid levels. Instead, our data indicated that the rs3746444 polymorphism may be a cardiovascular protective factor since this polymorphism significantly decreased the TG levels in CVD patients (Table 4).

Subgroup analyses by gender, ethnicity, and health status were performed since they might be important environmental factors to determine associative risk with lipid metabolism. For instance, the present meta-analysis indicated that gender might modulate the associations of the rs2910164 polymorphism with lipid levels since the significant associations were only observed in females (Table 1). Moreover, ethnicity might also modulate the associations of the rs2910164 polymorphism with lipid levels due to a stronger association which was observed in Caucasians, but not in Asians (Tables 1, 2).

According to the 2018 ACC/AHA (Grundy et al., 2019), the 2019 ESC/EAS (Mach et al., 2020), and the adult treatment panel III (ATP III) cholesterol guidelines (National Cholesterol Education Program (NCEP) Expert Panel on Detection, 2002), LDL-C was considered as the major cause of CAD and treated as the primary target for therapy, while other lipids were used as the secondary or supplementary therapeutic targets. In the present study, the rs2910164 polymorphism showed a relatively strong correlation with LDL-C levels in preliminary analysis (SMD = 0.37, 95% CI = 0.11–0.63, *p* = 0.01). In addition, sensitivity analysis further strengthened its effect on LDL-C levels (SMD = 0.45, 95% CI = 0.13–0.77, *p* = 0.01). It indicated that the rs2910164 polymorphism was robustly correlated with LDL-C levels. However, the correlation between the rs2910164 polymorphism and LDL-C levels only showed a marginal significance (SMD = 0.08, 95% CI = −0.01–0.17, *p* = 0.08) after excluding the studies with heterogeneity (Table 2). By using the Galbraith plot, the heterogeneity was primarily from two studies by Mehanna et al. (2015) and Abo-Elmatty and Mehanna (2019), in which the classification of populations was both Egyptian and females; it indicated that ethnicities and gender may contribute to the heterogeneity between the rs2910164 polymorphism and LDL-C levels. Together, our study showed that the rs2910164 polymorphism of the *miRNA-146a* gene was robustly associated with LDL-C levels; despite the heterogeneity, future population-based multicenter studies are needed to clarify or verify our findings.

Several limitations of the present meta-analysis should be noted. First of all, dyslipidemia is involved in a large number of genes as well as some environmental factors. However, the interactions of the rs2910164 polymorphism with other polymorphic loci or environmental factors on plasma lipid levels have not been investigated in this meta-analysis due to the lack of original data from the included studies. Secondly, a relatively small number of individuals were included in the lipid association analysis for rs2910164 and rs3746444 due to the limited number of studies that met the inclusion criteria, which may reduce the statistic power and even cause type I error (false-positive results). Thirdly, this meta-analysis only included the studies published in English and Chinese as it is very difficult to get the full papers published in various languages.

## Conclusions

The *miRNA-146a* rs2910164 polymorphism is significantly associated with atherogenic dyslipidemia.

## Data Availability Statement

The datasets presented in this study can be found in online repositories. The names of the repository/repositories and accession number(s) can be found in the article/Supplementary Material.

## Author Contributions

ZL conceived and designed this study as well as drafted the manuscript. ZL, FL, and SW carried out the searches and collected the data. ZL and FL performed the statistical analyses. ZL and FL were responsible for revising the manuscript critically for important intellectual contents. All authors reviewed and approved the final manuscript.

## Conflict of Interest

The authors declare that the research was conducted in the absence of any commercial or financial relationships that could be construed as a potential conflict of interest.

## Publisher's Note

All claims expressed in this article are solely those of the authors and do not necessarily represent those of their affiliated organizations, or those of the publisher, the editors and the reviewers. Any product that may be evaluated in this article, or claim that may be made by its manufacturer, is not guaranteed or endorsed by the publisher.
