# Supplementary material for: Associations of the miRNA-146a rs2910164 and the miRNA-499a rs3746444 Polymorphisms With Plasma Lipid Levels: A Meta-Analysis
Source: Front Genet. 2021 Sep 27;12:746686. doi: 10.3389/fgene.2021.746686 (PMC8503190; doi:10.3389/fgene.2021.746686)
Supplement: Supplementary file 1 [file Data_Sheet_1.docx]

Supplementary Material

**Supplemental Tables**

**STable 1.** Characteristics of the studies included in the meta-analysis.

**STable 2.** Plasma lipid levels by the genotypes of *miRNA-146a* rs2910164 polymorphism.

**STable 3.** Plasma lipid levels by the genotypes of *miRNA-499a* rs3746444 polymorphism.

**Supplemental Figures:**

**SFig 1.** Forest plot of the meta-analysis between *miRNA-499a* rs3746444 polymorphism and plasma TG levels.

**SFig 2.** Forest plot of the meta-analysis between *miRNA-499a* rs3746444 polymorphism and plasma TC levels.

**SFig 3.** Forest plot of the meta-analysis between *miRNA-499a* rs3746444 polymorphism and plasma LDL-C levels.

**SFig 4.** Forest plot of the meta-analysis between *miRNA-499a* rs3746444 polymorphism and plasma HDL-C levels.

**SFig 5.** Sensitivity analysis between *miRNA-499a* rs3746444 polymorphism and plasma TC levels.

**SFig 6.** Sensitivity analysis between *miRNA-499a* rs3746444 polymorphism and plasma LDL-C levels.

**SFig 7.** Sensitivity analysis between *miRNA-499a* rs3746444 polymorphism and plasma HDL-C levels.

**SFig 8.** Sensitivity analysis between *miRNA-146a* rs2910164 polymorphism and plasma TC levels.

**SFig 9.** Sensitivity analysis between *miRNA-146a* rs2910164 polymorphism and plasma LDL-C levels.

**SFig 10.** Sensitivity analysis between *miRNA-146a* rs2910164 polymorphism and plasma HDL-C levels.

**SFig 11.** Sensitivity analysis between *miRNA-499a* rs3746444 polymorphism and plasma TG levels.

**SFig 12.** Sensitivity analysis between *miRNA-146a* rs2910164 polymorphism and plasma TG levels.

**SFig 13.** Risk bias plot of the meta-analysis between *miRNA-499a* rs3746444 polymorphism and plasma lipid levels.

**SFig 14.** Begg’s funnel plot of the association analysis between *miRNA-146a* rs2910164 polymorphism and plasma TC levels.

**SFig 15.** Begg’s funnel plot of the association analysis between *miRNA-146a* rs2910164 polymorphism and plasma LDL-C levels.

**SFig 16.** Begg’s funnel plot of the association analysis between *miRNA-146a* rs2910164 polymorphism and plasma HDL-C levels.

**SFig 17.** Begg’s funnel plot of the association analysis between *miRNA-499a* rs3746444 polymorphism and plasma TG levels.

**SFig 18.** Begg’s funnel plot of the association analysis between *miRNA-499a* rs3746444 polymorphism and plasma TC levels.

**SFig 19.** Begg’s funnel plot of the association analysis between *miRNA-499a* rs3746444 polymorphism and plasma LDL-C levels.

**SFig 20.** Begg’s funnel plot of the association analysis between *miRNA-499a* rs3746444 polymorphism and plasma HDL-C levels

**STable 1.** Characteristics of the studies included in the meta-analysis.

| **First author [reference]** | **Polymorphisms** | **Year** | **Study population** | **Ethnicity** | **Gender** | **Outcomes** | **Health status** | **Study summary** |
| --- | --- | --- | --- | --- | --- | --- | --- | --- |
| Li QZ [R1] | rs2910164, rs3746444 | 2015 | Chinese population | Asian | M/F | TG, TC, LDL-C, HDL-C | Patients with CAD | Li et al. investigated the effect of rs2910164 and rs3746444 polymorphisms on prognosis in 1004 Chinese Han patients with CAD, and found that rs3746444 polymorphism was associated with even-free survival therefore concluded that rs3746444 polymorphism might be a potential biomarker for the clinical prognosis of CAD. |
| Mehanna ET [R2] | rs2910164 | 2015 | Egyptian population | Caucasian | F | TG, TC, LDL-C, HDL-C | Patients with MetS and control subjects | Mehanna et al. assessed the relation of rs2910164 polymorphism with MetS and its component traits in 200 Egyptian women, they found that GC and CC genotypes were associated with increased waist circumference, BMI, TG, TC, LDL-C, systolic and diastolic blood pressure and thus concluded that the C allele of miRNA-146a rs2910164 was associated with increased susceptibility to MetS and its phenotypes. |
| Alipoor B [R3] | rs2910164 | 2016 | Iranian population | Caucasian | M/F | TG, TC, LDL-C, HDL-C | Patients with T2DM and control subjects | Alipoor et al. explored the association between rs2910164 polymorphism with the susceptibility to T2DM and its related metabolic traits in 375 Iranian, and found that the CC genotype was associated with increased TG, TC, diastolic blood pressure, fasting blood glucose and HbAlc levels therefore concluded that rs2910164 polymorphism might be associated with T2DM and its cardiovascular risk factors. |
| Chen LB [R4] | rs3746444 | 2017 | Chinese population | Asian | M/F | HDL-C | Healthy subjects | Chen et al. explored whether rs3746444 polymorphism was associated with the HDL level in 46 healthy Chinese Han subjects, and found that the HDL level in AA genotype was significantly lower than those in AG and GG genotype. |
| Luo HC [R5] | rs3746444 | 2017 | Chinese population | Asian | M/F | TG, TC, LDL-C, HDL-C | Patients with IS | Luo et al. investigated the genetic polymorphisms of miR-146a and miR-499 and genetic susceptibility of IS in 601 Chinese Han subjects, and found that the genetic polymorphism of miR-499 rs3746444 may increase the risk of IS by increasing LDL-C and VLDL-C levels. |
| Chen CM [R6] | rs2910164 | 2018 | Chinese population | Asian | M/F | TG, TC, LDL-C, HDL-C | Patients with CAD and control subjects | Chen et al. investigated the association between rs2910164 polymorphism and ACS in a southern Chinese Han population, they found that C allele of rs2910164 polymorphism significantly increased TC and LDL-C levels  in ACS patients and concluded that rs2910164 polymorphism largely increased susceptibility of ACS. |
| Ciccacci C [R7] | rs3746444 | 2018 | Italian population | Caucasion | M/F | TG, TC, LDL-C, HDL-C | Patients with T2DM | Ciccacci et al. investigated whether rs3746444 polymorphism was associated with susceptibility of diabetic polyneuropathy and cardiovascular autonomic neuropathy in 150 patients with T2DM, and found that GG genotype was associated with a higher risk of developing diabetic polyneuropathy and cardiovascular autonomic neuropathy therefore concluded that the patients carrying the rs3746444 GG genotype had a higher risk of cardiovascular autonomic neuropathy development. |
| Abo-Elmatty DM [R8] | rs2910164 | 2019 | Egyptian population | Caucasion | F | TG, TC, LDL-C, HDL-C | Patients with GD and preeclampsia | Abo-Elmatty et al. investigated the frequency of rs2910164 polymorphism and its possible correlation with the incidence of preeclampsia in 250 GD patients, and found that the frequency of the C allele of rs2910164 polymorphism was significantly higher among patients of GD combined with preeclampsia compared to the control subjects therefore  concluded that the CC genotype of rs2910164 polymorphism may be related to increased incidence of preeclampsia in GD patients. |
| Qiu XY [R9] | rs3746444 | 2019 | Chinese population | Asian | M/F | TG, TC, LDL-C, HDL-C | Patients with AF and control subjects | Qiu et al. evaluated the rs2910164 and rs3746444 polymorphisms and their putative association with inflammatory markers in 65 Chinese Han patients with AF, and found that the C allele of rs3746444 had higher plasma levels of IL-6 and hs-CRP than did patients with the T allele. |
| Qiu H1 [R10] | rs2910164, rs3746444 | 2020 | Chinese population | Asian | M/F | TG, TC, LDL-C, HDL-C | Patients with CAD | Qiu et al. identified the correlation of miR SNPs with the susceptibility to CAD in 1614 Chinese Han subjects, and found that rs11614913 polymorphism decreased the susceptibility of CAD in female subjects. |
| Qiu H2 [R10] | rs2910164, rs3746444 | 2020 | Chinese population | Asian | M/F | TG, TC, LDL-C, HDL-C | Healthy subjects |  |

*MiRNA*: microRNA gene; M: male; F: female; CAD: coronary artery disease; MI: myocardial infarction; ACS: acute coronary syndrome; MetS: metabolic syndrome; T2DM: type 2 diabetes mellitus; IS: ischemic stroke; GD: gestational diabetes; BMI: body mass index; TG: triglycerides; AF: atrial fibrillation; TC: total cholesterol; LDL-C: low-density lipoprotein cholesterol; HDL-C: high-density lipoprotein cholesterol.

**STable 2.** Plasma lipid levels by the genotypes of *miRNA-146a* rs2910164 polymorphism.

| **First author, reference** | **Number** | |  | **TG, mmol/L** | |  | **TC, mmol/L** | |  | **LDL-C, mmol/L** | |  | **HDL-C, mmol/L** | |
| --- | --- | --- | --- | --- | --- | --- | --- | --- | --- | --- | --- | --- | --- | --- |
|  | **CC** | **CG+GG** |  | **CC** | **CG+GG** |  | **CC** | **CG+GG** |  | **CC** | **CG+GG** |  | **CC** | **CG+GG** |
| Li et al. [R1] | 343 | 661 |  | 1.7±0.9 | 2.04±1.27 |  | 4.6±1.1 | 4.7±1.03 |  | 2.7±0.9 | 2.73±0.83 |  | 1.2±0.3 | 1.2±0.4 |
| Mehanna et al. [R2] | 76 | 124 |  | 1.59±0.51 | 1.94±0.78 |  | 4.62±0.92 | 5.43±1.21 |  | 2.47±0.92 | 3.20±1.14 |  | 1.39±0.33 | 1.27±0.28 |
| Alipoor et al. [R3] | 204 | 171 |  | 1.57±0.8 | 1.85±0.73 |  | 4.18±1.17 | 4.46±1.06 |  | 2.24±0.56 | 2.33±0.71 |  | 1.14±0.25 | 1.06±0.26 |
| Chen et al. [R6] | 76 | 124 |  | 1.62±0.52 | 1.98±0.80 |  | 4.63±0.93 | 5.44±1.21 |  | 2.47±0.92 | 3.21±1.15 |  | 1.39±0.33 | 1.28±0.28 |
| Abo-Elmatty et al. [R8] | 37 | 48 |  | 2.04±0.38 | 2.45±0.39 |  | 6.48±0.39 | 7.29±0.64 |  | 4.15±0.59 | 4.95±0.61 |  | 1.3±0.21 | 1.09±0.18 |
| Qiu et al. [R10] | 60 | 440 |  | 1.72±1.24 | 1.71±1.17 |  | 4.37±1.42 | 4.25±1.19 |  | 2.84±1.25 | 2.7±1.07 |  | 0.99±0.28 | 1.03±0.3 |
| Qiu et al. [R10] | 154 | 952 |  | 1.59±1.04 | 1.61±1.01 |  | 4.68±1.05 | 4.86±1.05 |  | 2.95±0..84 | 3.11±0.86 |  | 1.29±0.34 | 1.22±0.36 |

*MiRNA*: microRNA gene; TG: triglycerides; TC: total cholesterol; LDL-C: low-density lipoprotein cholesterol; HDL-C: high-density lipoprotein cholesterol.

**STable 3.** Plasma lipid levels by the genotypes of *miRNA-499a* rs3746444 polymorphism.

| **First author, reference** | **Number** | |  | **TG, mmol/L** | |  | **TC, mmol/L** | |  | **LDL-C, mmol/L** | |  | **HDL-C, mmol/L** | |
| --- | --- | --- | --- | --- | --- | --- | --- | --- | --- | --- | --- | --- | --- | --- |
|  | **CC** | **CG+GG** |  | **CC** | **CG+GG** |  | **CC** | **CG+GG** |  | **CC** | **CG+GG** |  | **CC** | **CG+GG** |
| Li QZ [R1] | 668 | 336 |  | 2.1±1.1 | 1.88±1.2 |  | 4.8±0.8 | 4.7±1.1 |  | 2.7±0.7 | 2.72±0.9 |  | 1.1±0.3 | 1.12±0.4 |
| Chen LB [R4] | 22 | 24 |  | - | - |  | - | - |  | - | - |  | 1.2±0.25 | 1.73±0.24 |
| Luo HC [R5] | 215 | 83 |  | 1.87±1.04 | 1.75±1.18 |  | 5.07±1.21 | 5.21±1.04 |  | 2.98±2.08 | 3.03±0.81 |  | 1.22±0.30 | 1.25±0.31 |
| Ciccacci C [R7] | 84 | 66 |  | 1.69±1.22 | 1.92±3.83 |  | 4.5±0.92 | 4.35±1.13 |  | - | - |  | 1.23±0.4 | 1.15±0.29 |
| Qiu XY [R9] | 74 | 49 |  | 1.37±0.70 | 1.43±0.75 |  | 4.11±0.83 | 4.25±0.88 |  | 2.39±0.69 | 2.60±0.74 |  | 1.25±0.37 | 1.26±0.35 |
| Qiu H1 [R10] | 357 | 143 |  | 1.65±1.01 | 1.86±1.52 |  | 4.23±1.19 | 4.34±1.29 |  | 2.70±1.08 | 2.75±1.11 |  | 1.04±0.29 | 1.01±0.33 |
| Qiu H2 [R10] | 800 | 306 |  | 1.62±1.02 | 1.59±1 |  | 4.85±1.03 | 4.8±1.11 |  | 3.09±0.85 | 3.06±0.89 |  | 1.24±0.37 | 1.20±0.35 |

*MiRNA*: microRNA gene; TG: triglycerides; TC: total cholesterol; LDL-C: low-density lipoprotein cholesterol; HDL-C: high-density lipoprotein cholesterol.


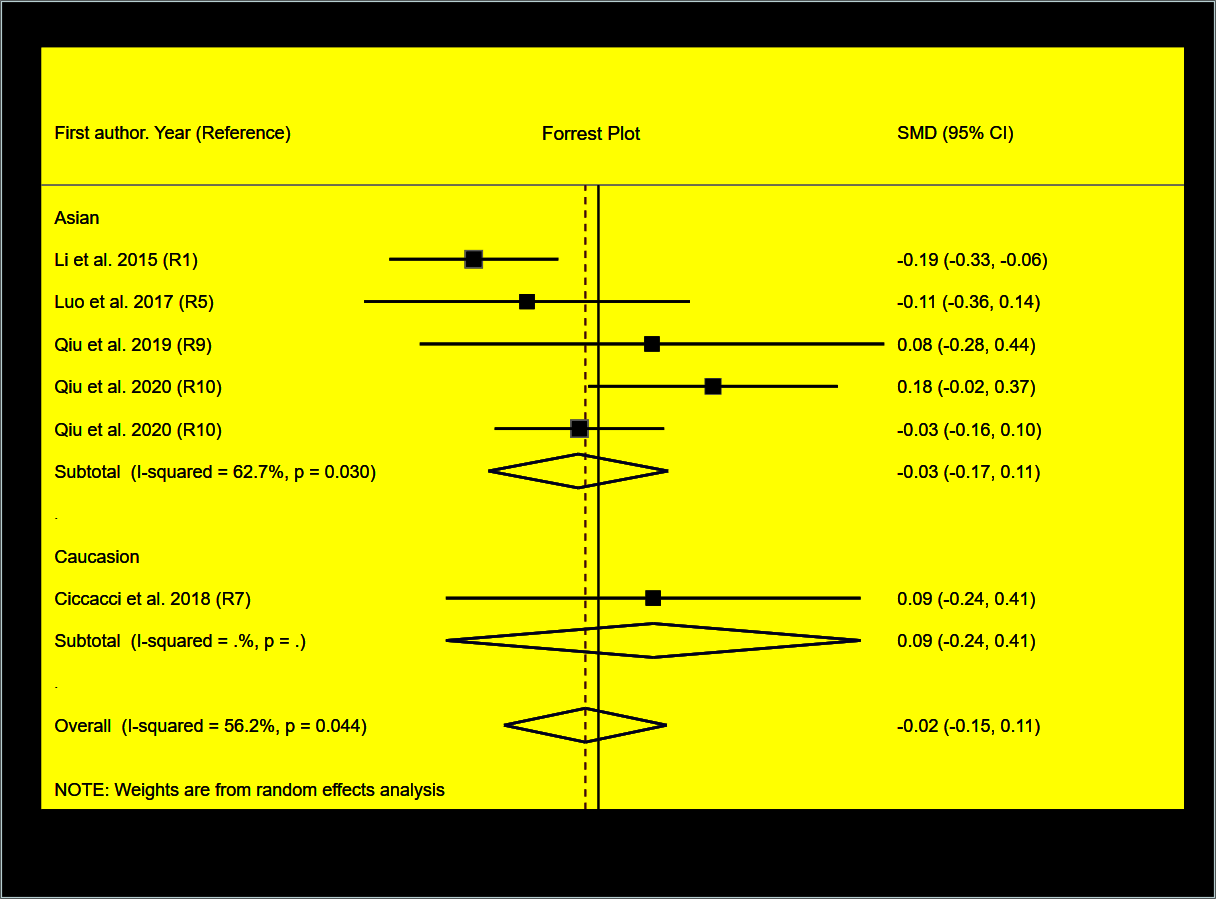
**SFig 1.** Forest plot of the meta-analysis between *miRNA-499a* rs3746444 polymorphism and plasma TG levels.


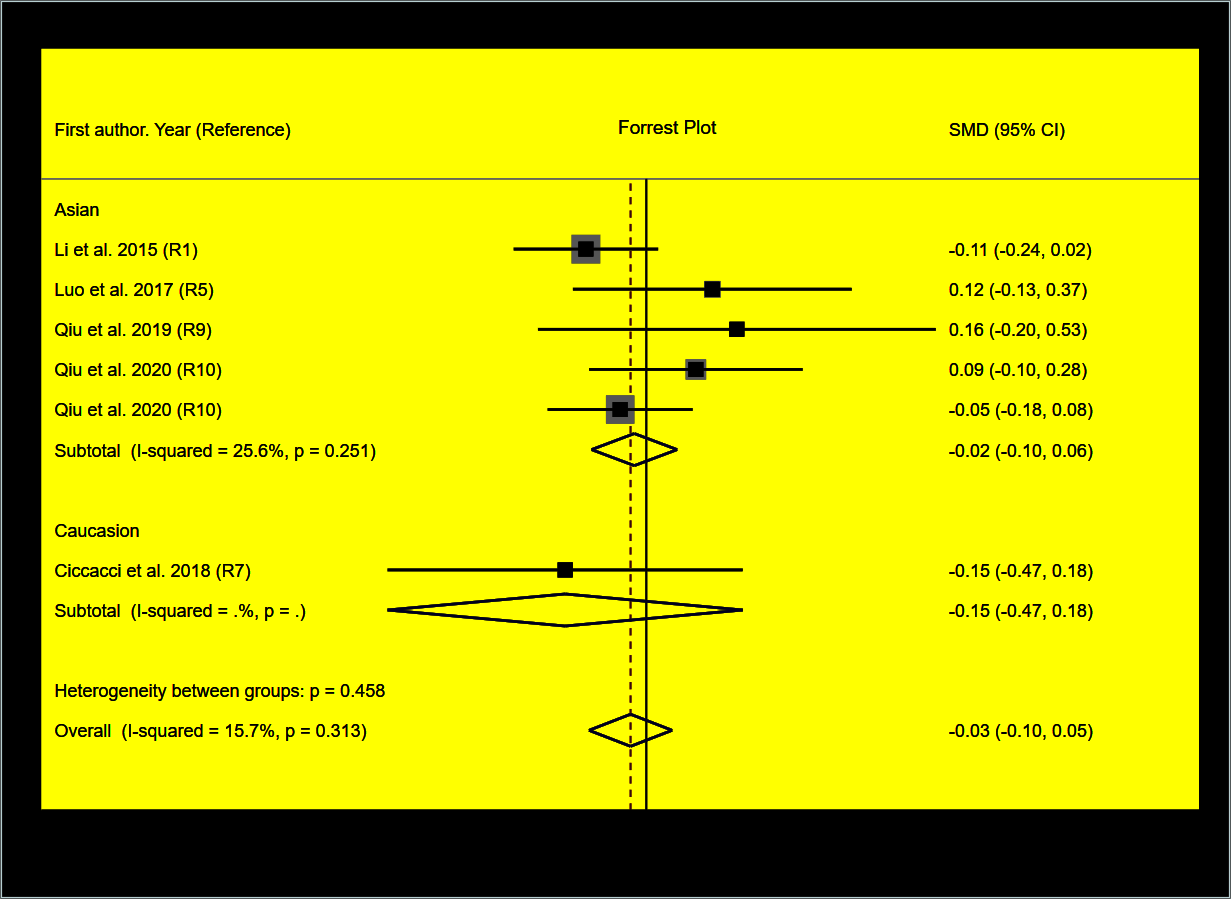


**SFig 2.** Forest plot of the meta-analysis between *miRNA-499a* rs3746444 polymorphism and plasma TC levels.


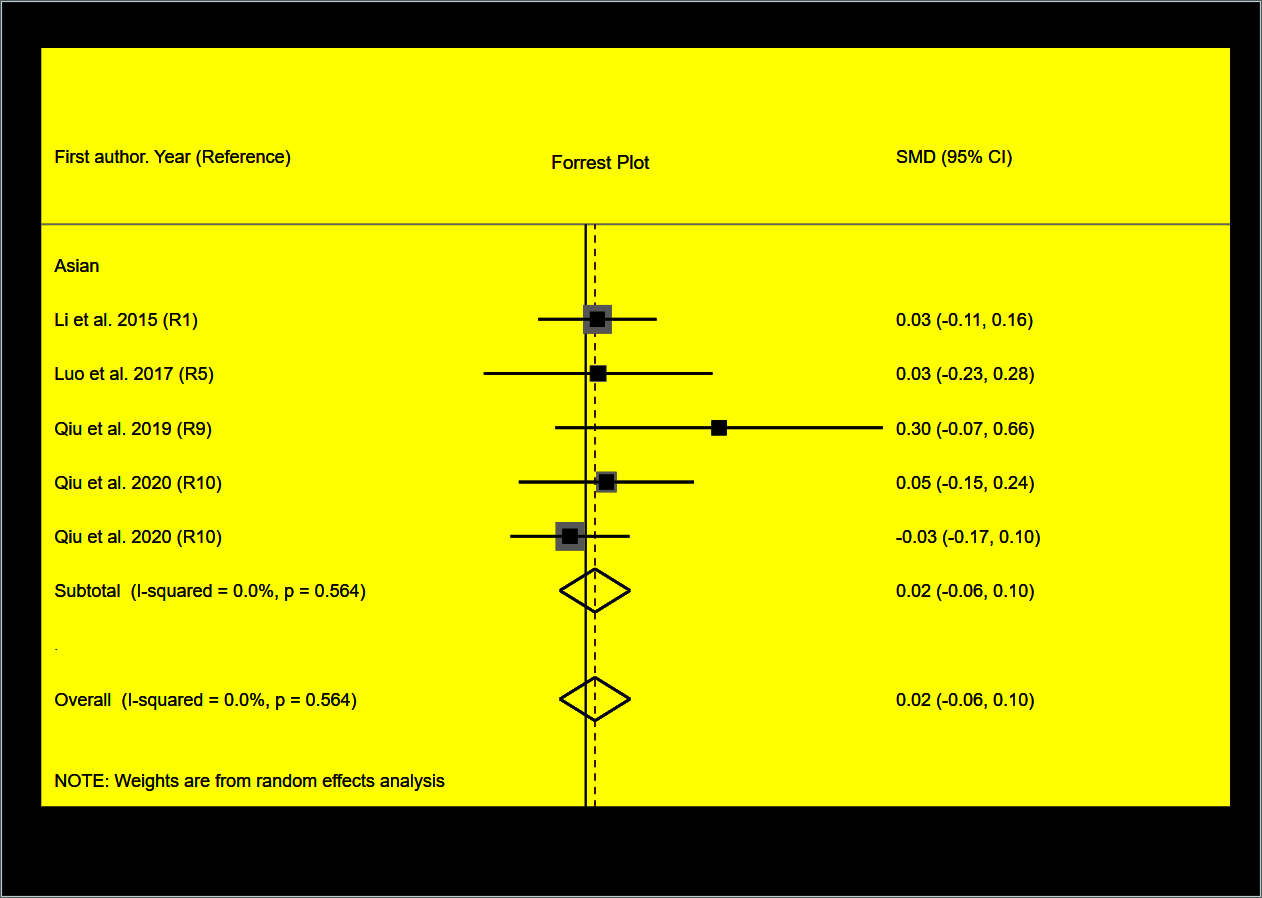
**SFig 3.** Forest plot of the meta-analysis between *miRNA-499a* rs3746444 polymorphism and plasma LDL-C levels.


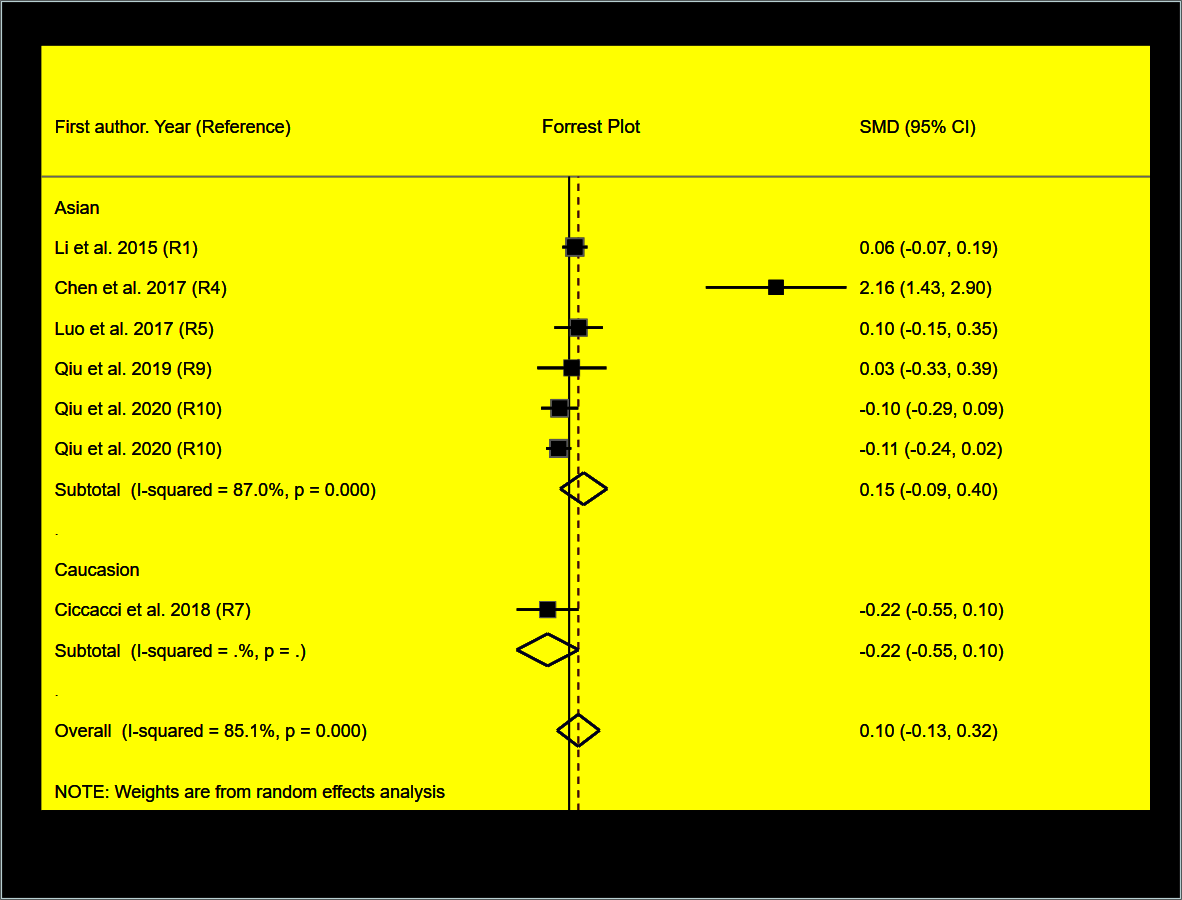
**SFig 4.** Forest plot of the meta-analysis between *miRNA-499a* rs3746444 polymorphism and plasma HDL-C levels.


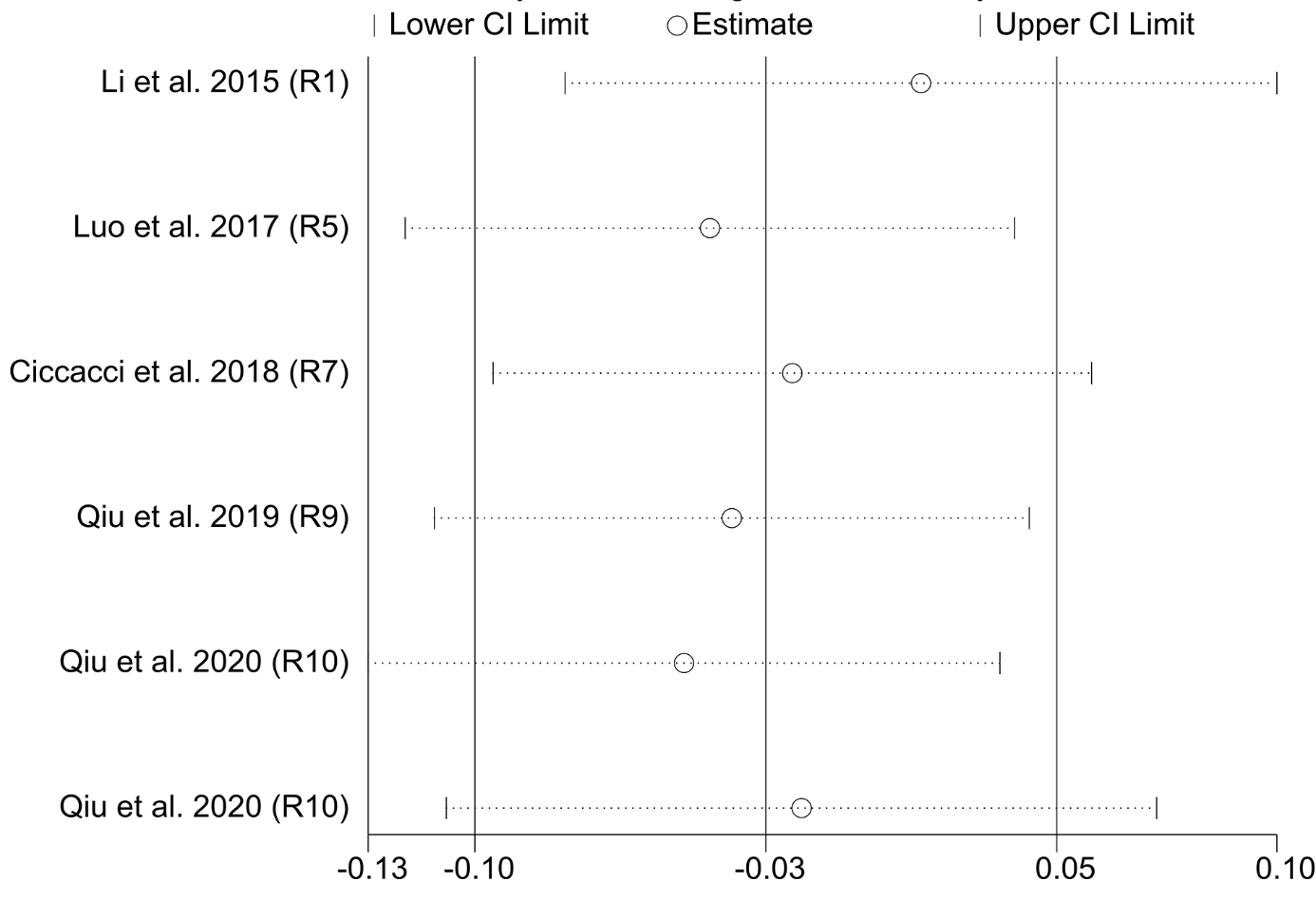


**SFig 5.** Sensitivity analysis between *miRNA-499a* rs3746444 polymorphism and plasma TC levels.


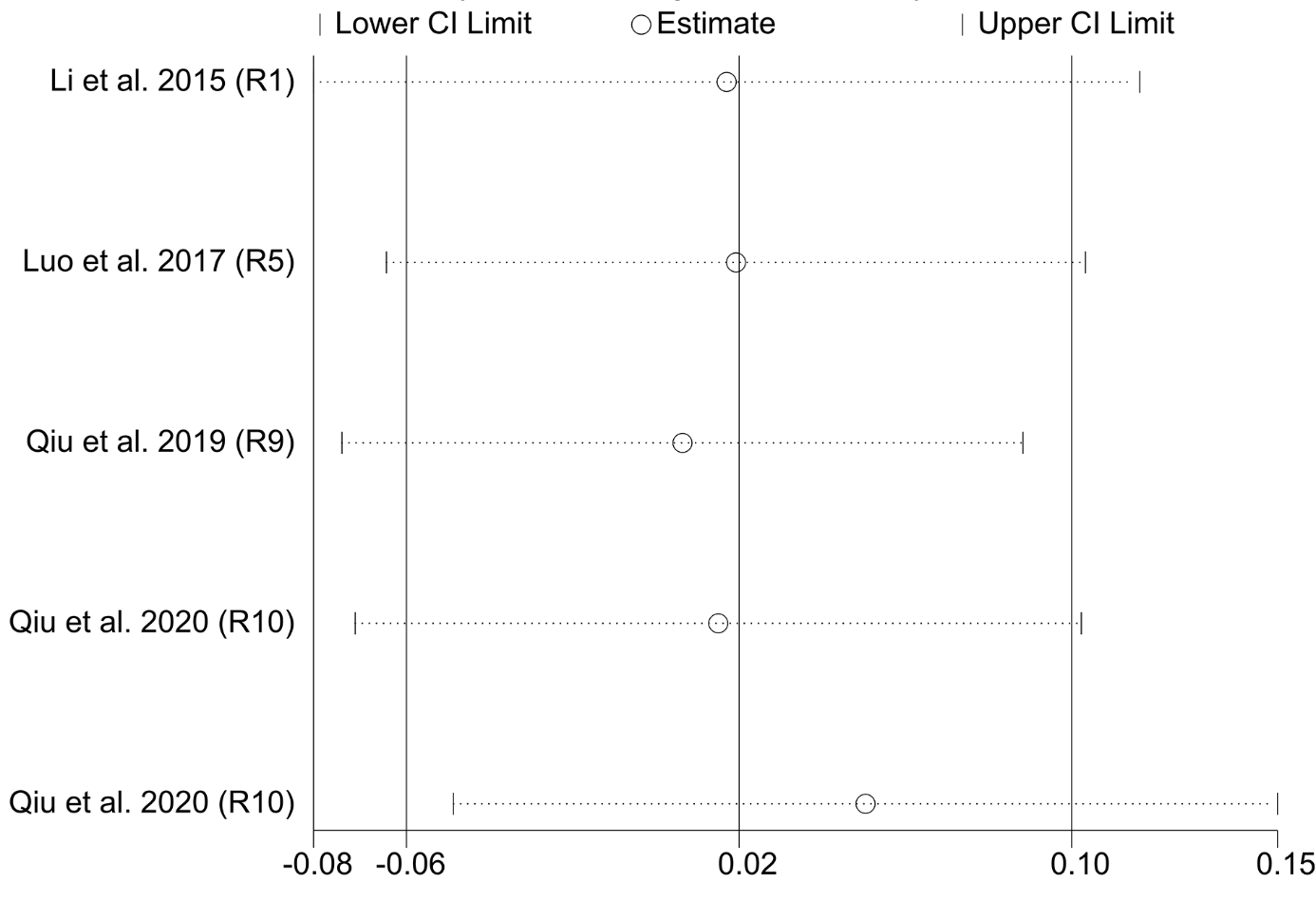


**SFig 6.** Sensitivity analysis between *miRNA-499a* rs3746444 polymorphism and plasma LDL-C levels.


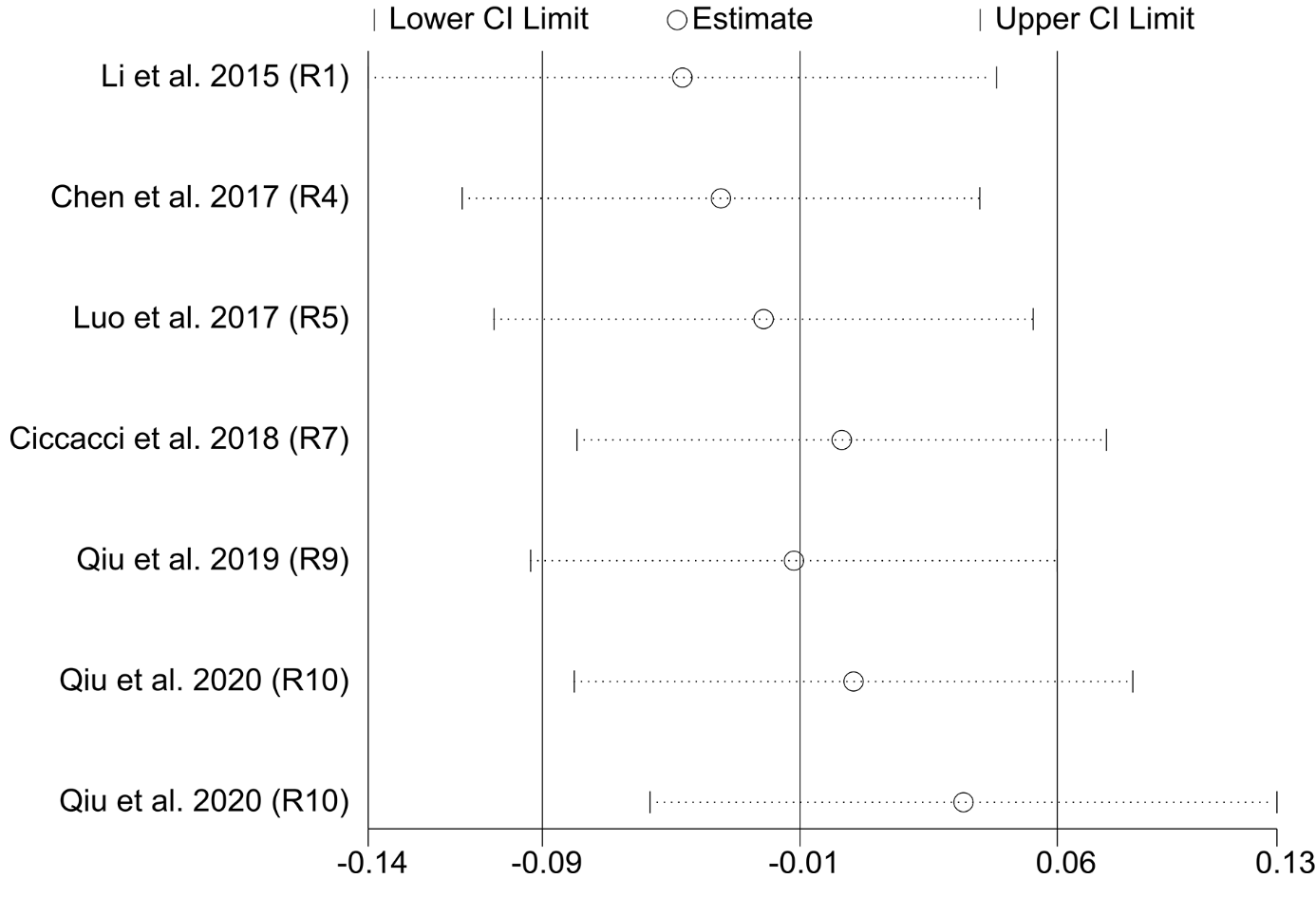


**SFig 7.** Sensitivity analysis between *miRNA-499a* rs3746444 polymorphism and plasma HDL-C levels.


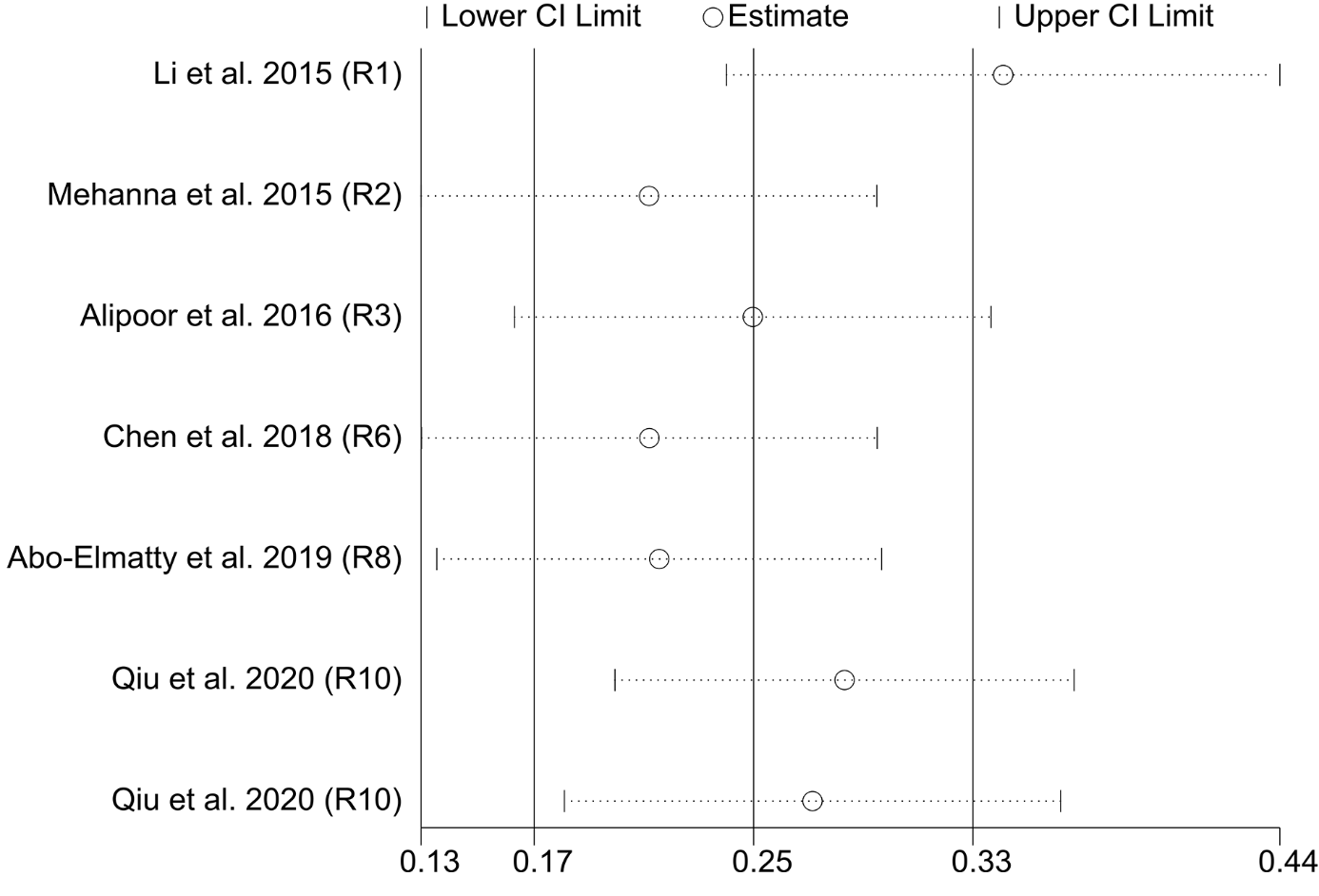


**SFig 8.** Sensitivity analysis between *miRNA-146a* rs2910164 polymorphism and plasma TC levels.


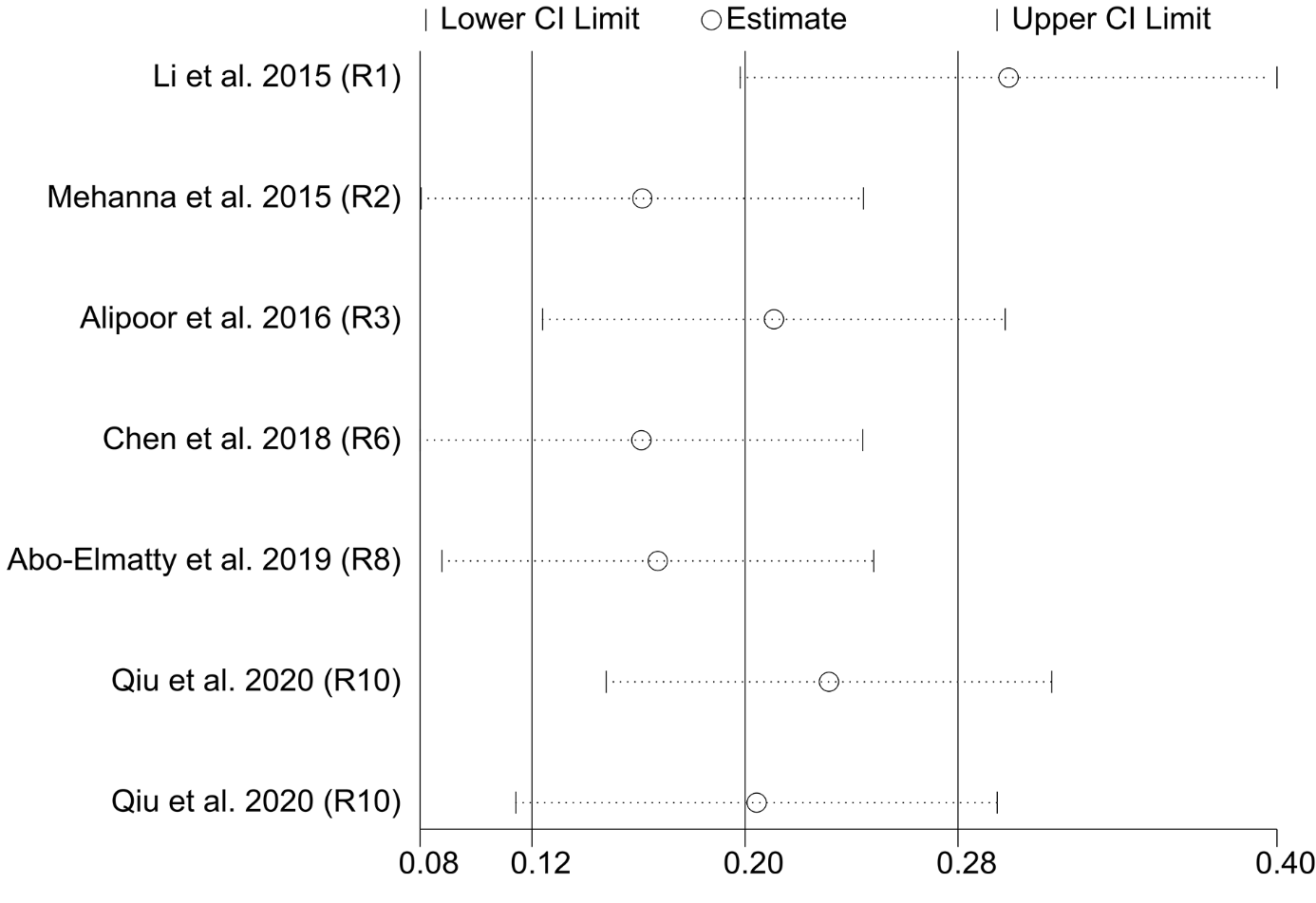


**SFig 9.** Sensitivity analysis between *miRNA-146a* rs2910164 polymorphism and plasma LDL-C levels.


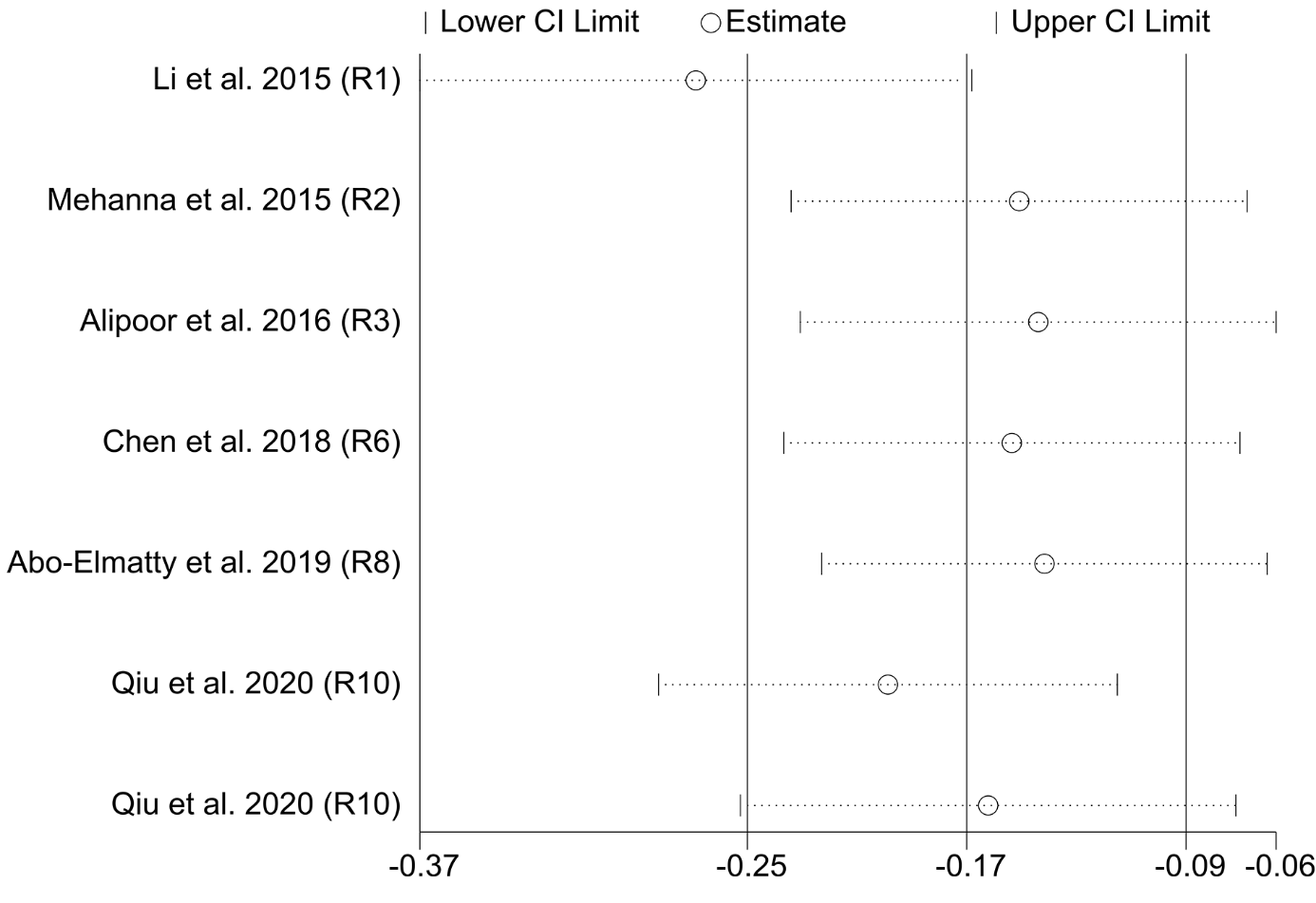


**SFig 10.** Sensitivity analysis between *miRNA-146a* rs2910164 polymorphism and plasma HDL-C levels.


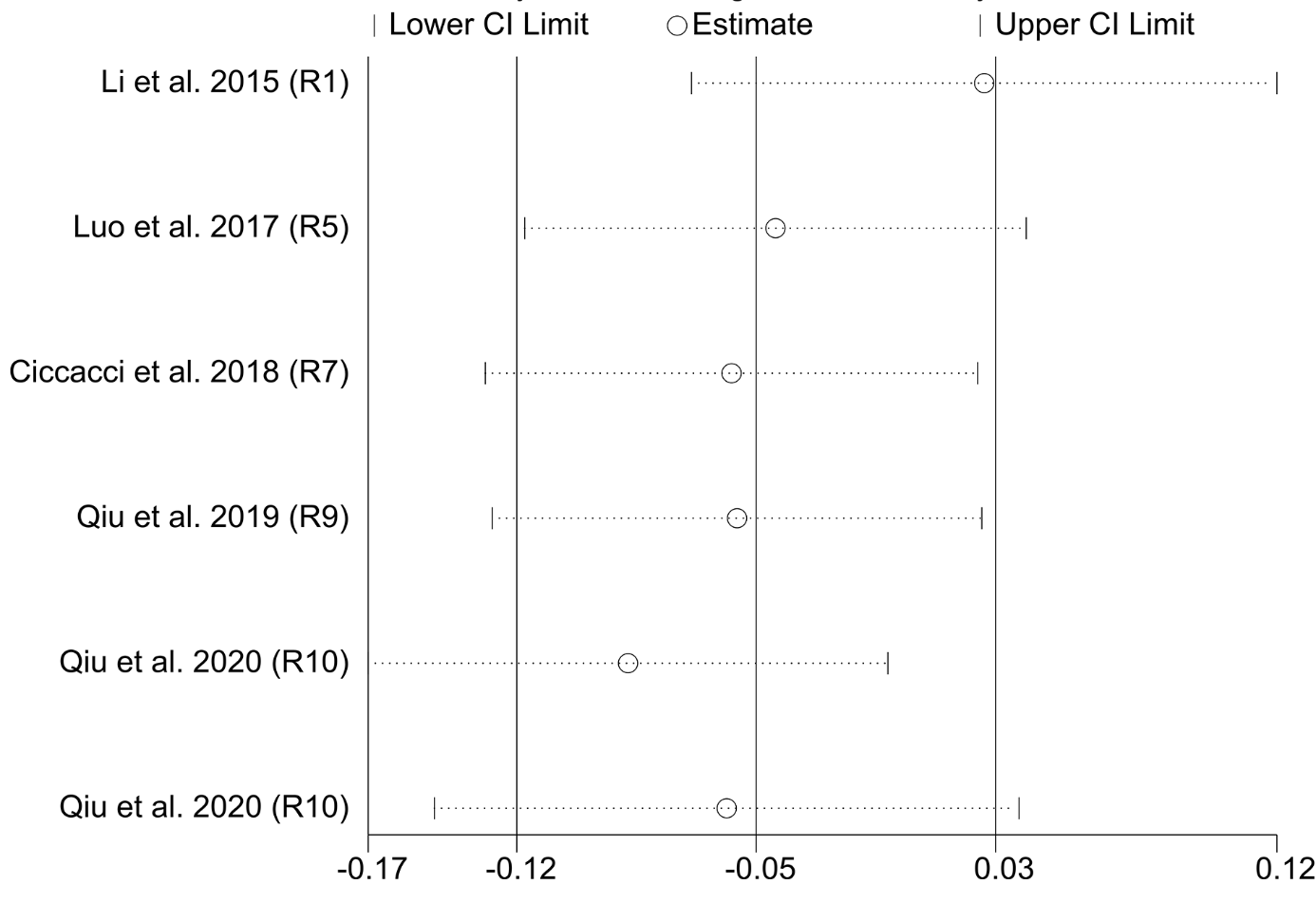


**SFig 11.** Sensitivity analysis between *miRNA-499a* rs3746444 polymorphism and plasma TG levels.


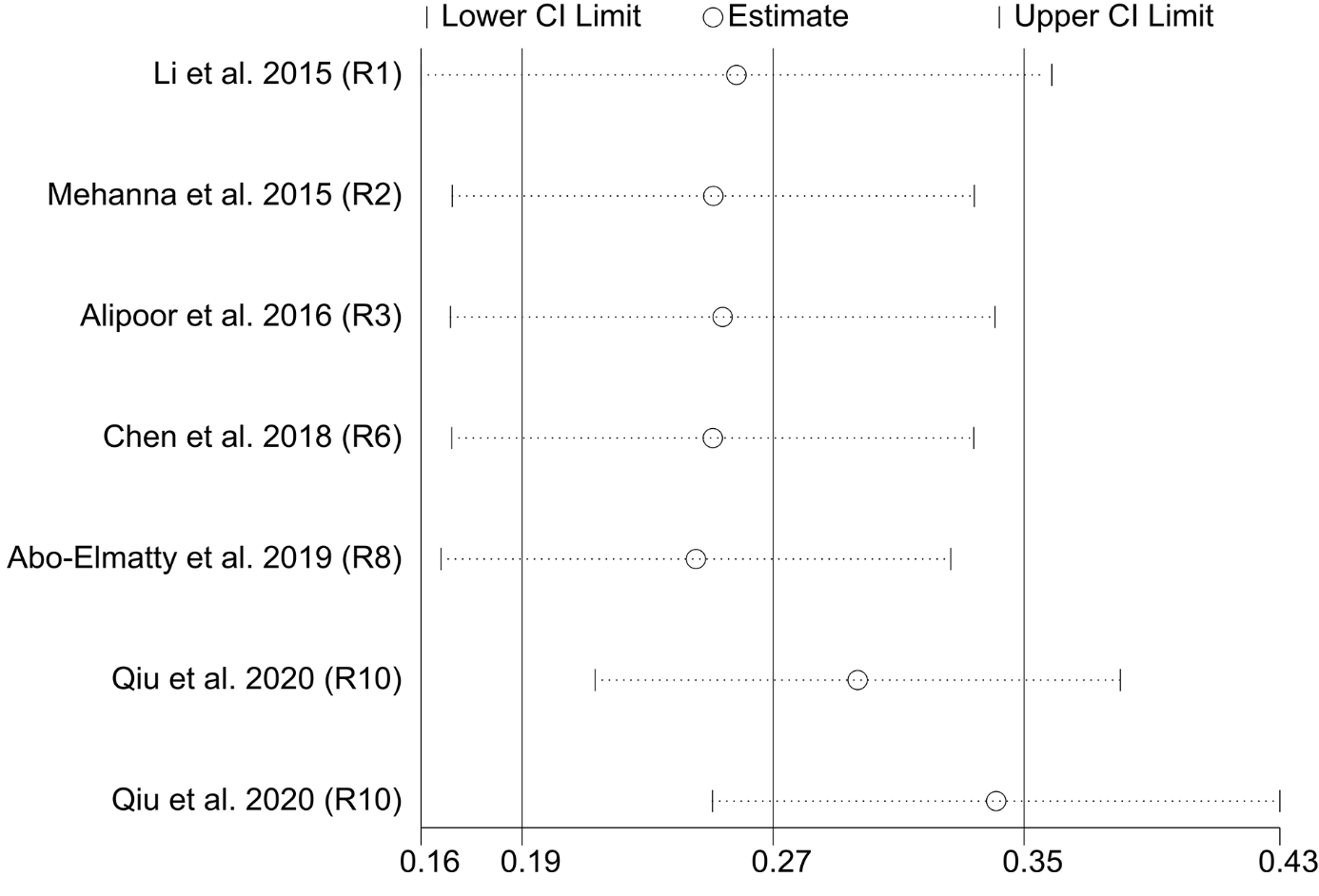


**SFig 12.** Sensitivity analysis between *miRNA-146a* rs2910164 polymorphism and plasma TG levels.


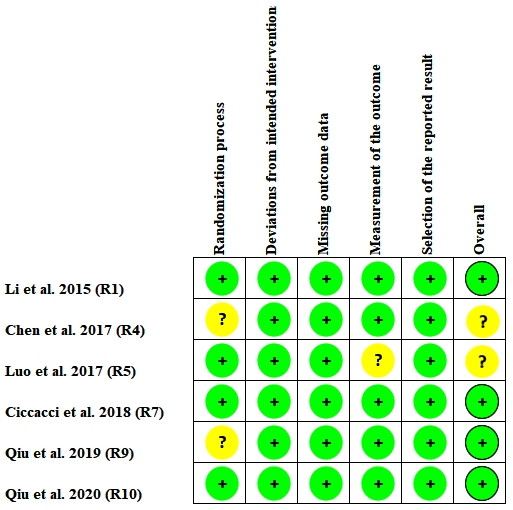


**SFig 13.** Risk bias plot of the meta-analysis between *miRNA-499a* rs3746444 polymorphism and plasma lipid levels (for assessment of each entry, green represents low risk of bias and yellow refers to unclear risk of bias).

.


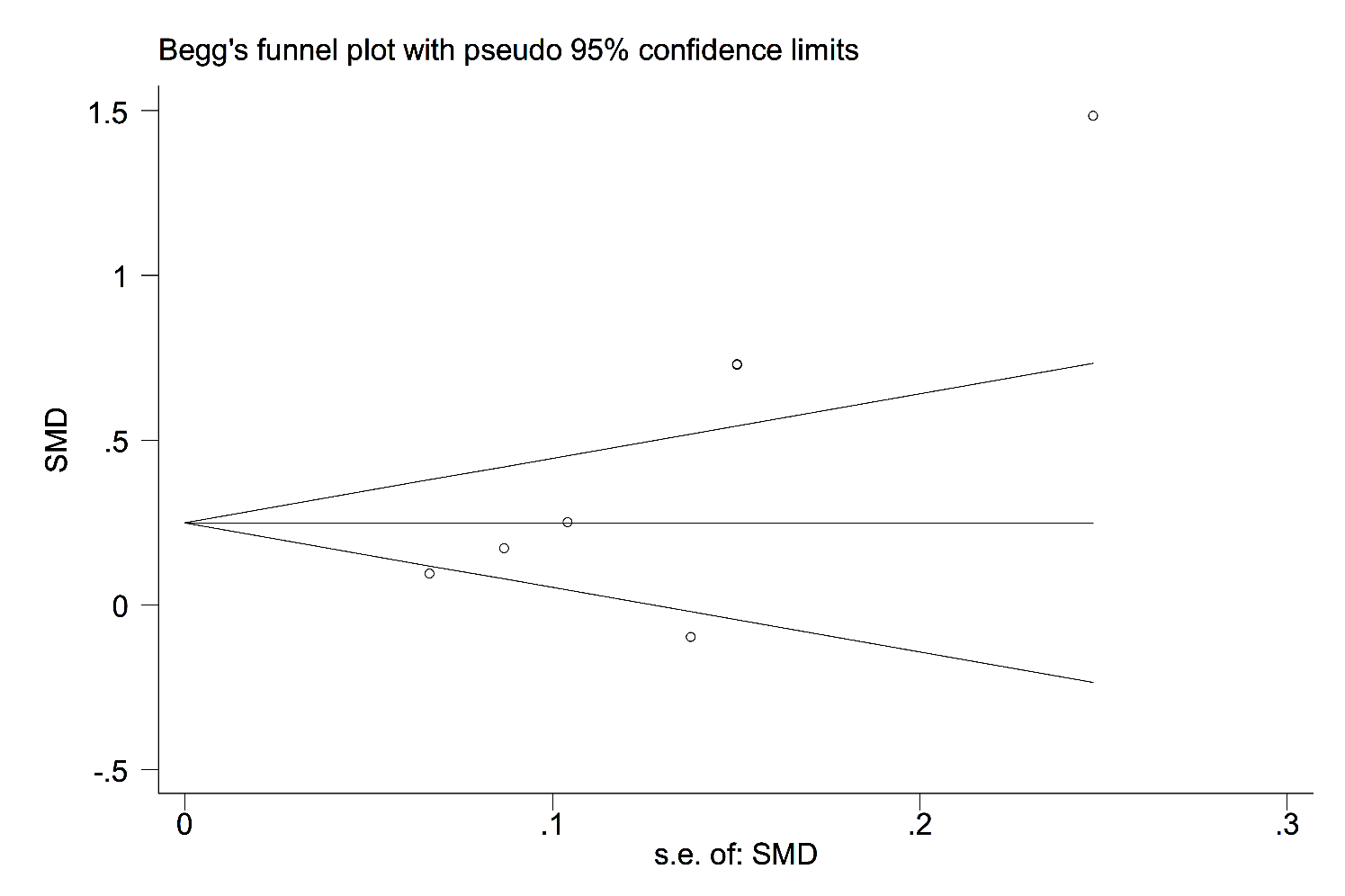
**SFig 14.** Begg’s funnel plot of the association analysis between *miRNA-146a* rs2910164 polymorphism and plasma TC levels.


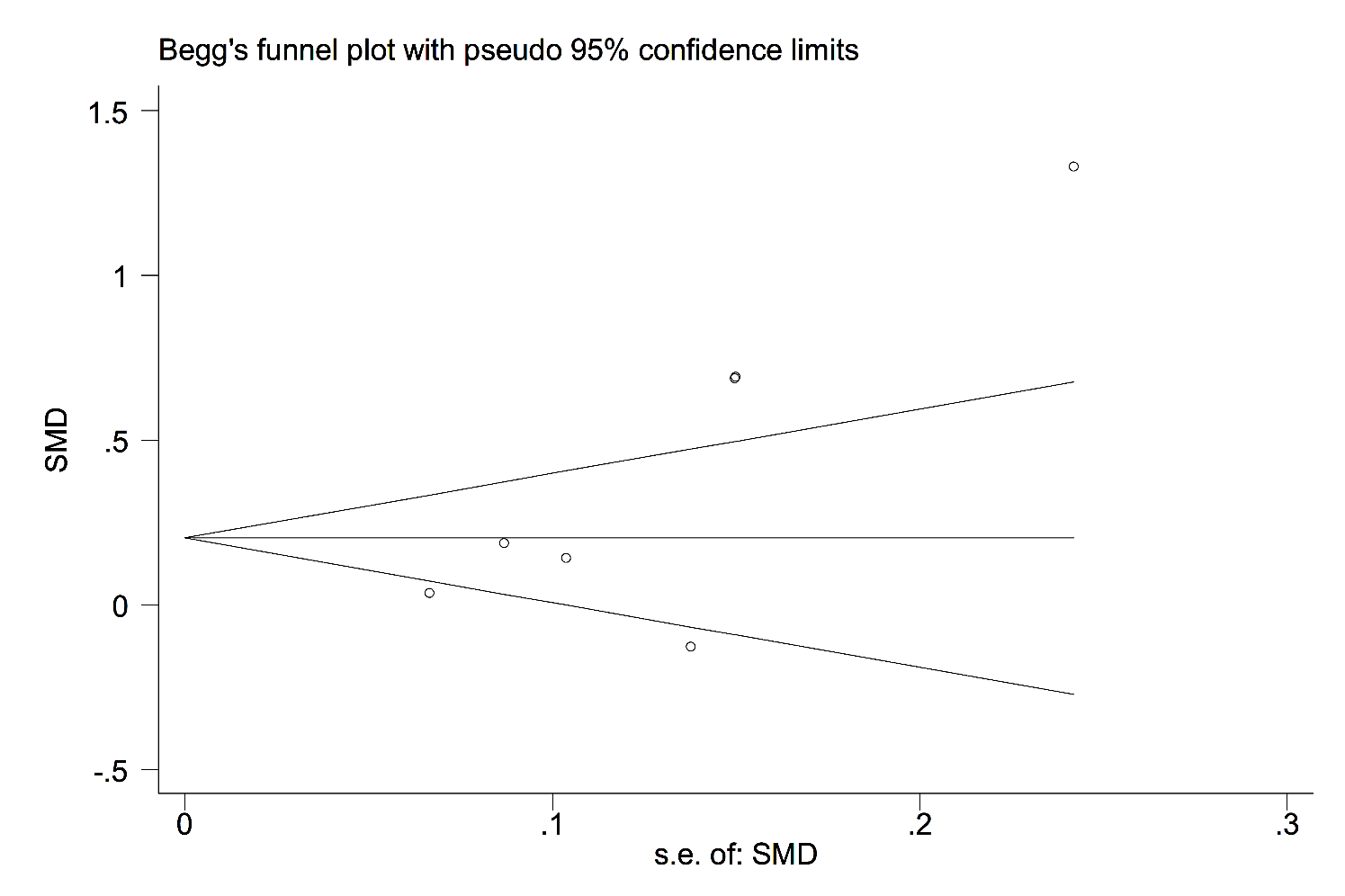
**SFig 15.** Begg’s funnel plot of the association analysis between *miRNA-146a* rs2910164 polymorphism and plasma LDL-C levels.


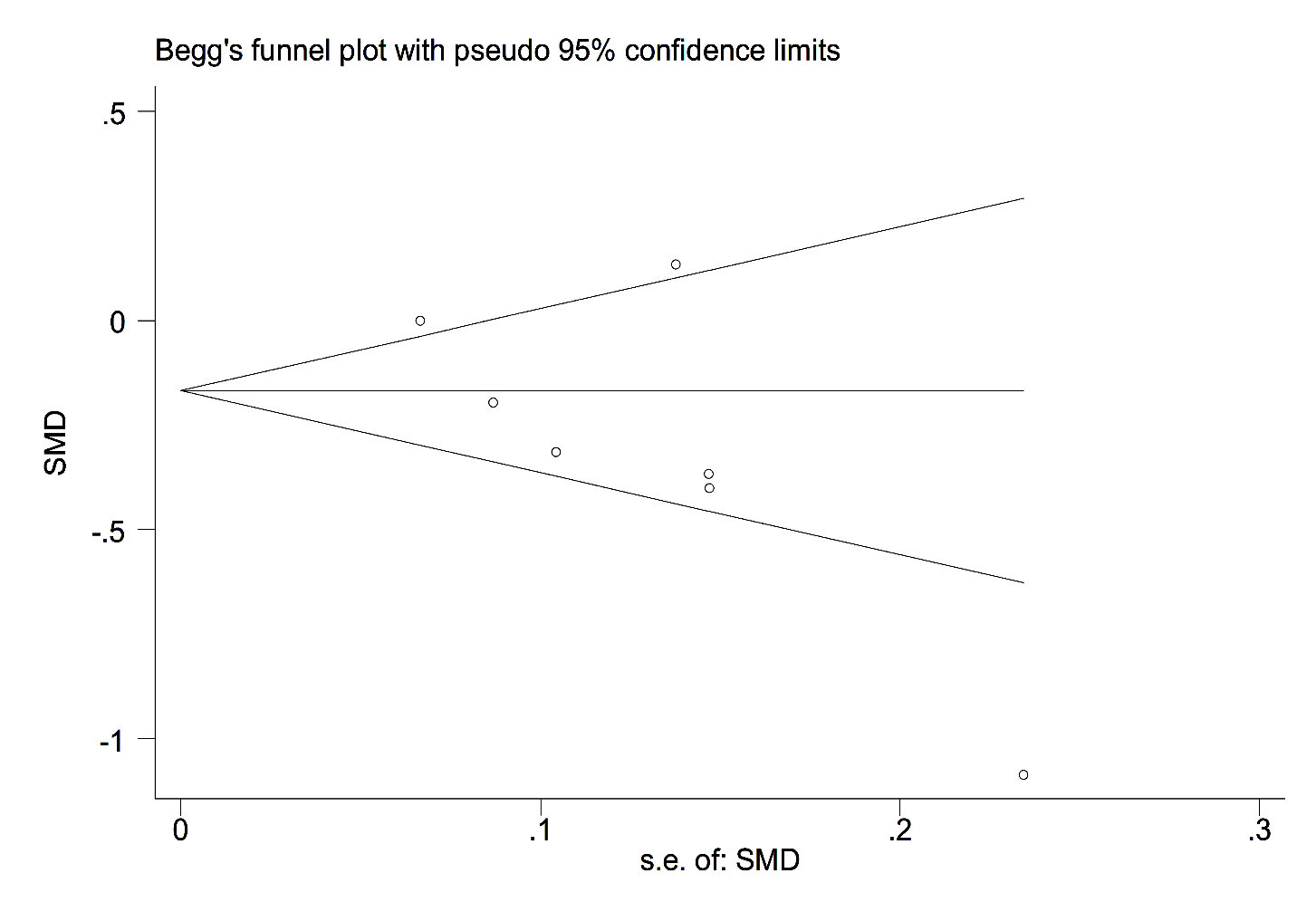
**SFig 16.** Begg’s funnel plot of the association analysis between *miRNA-146a* rs2910164 polymorphism and plasma HDL-C levels.


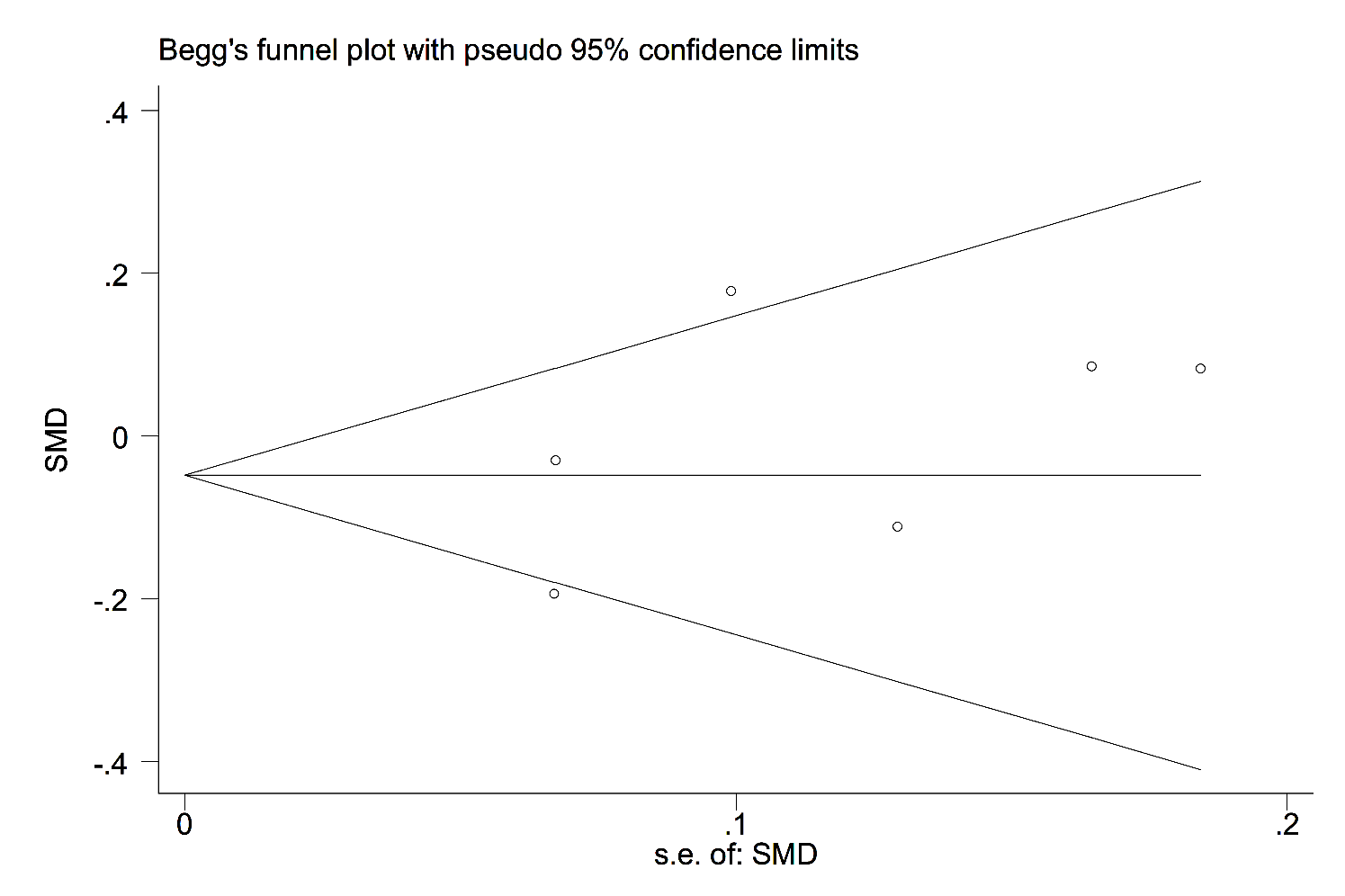
**SFig 17.** Begg’s funnel plot of the association analysis between *miRNA-499a* rs3746444 polymorphism and plasma TG levels.


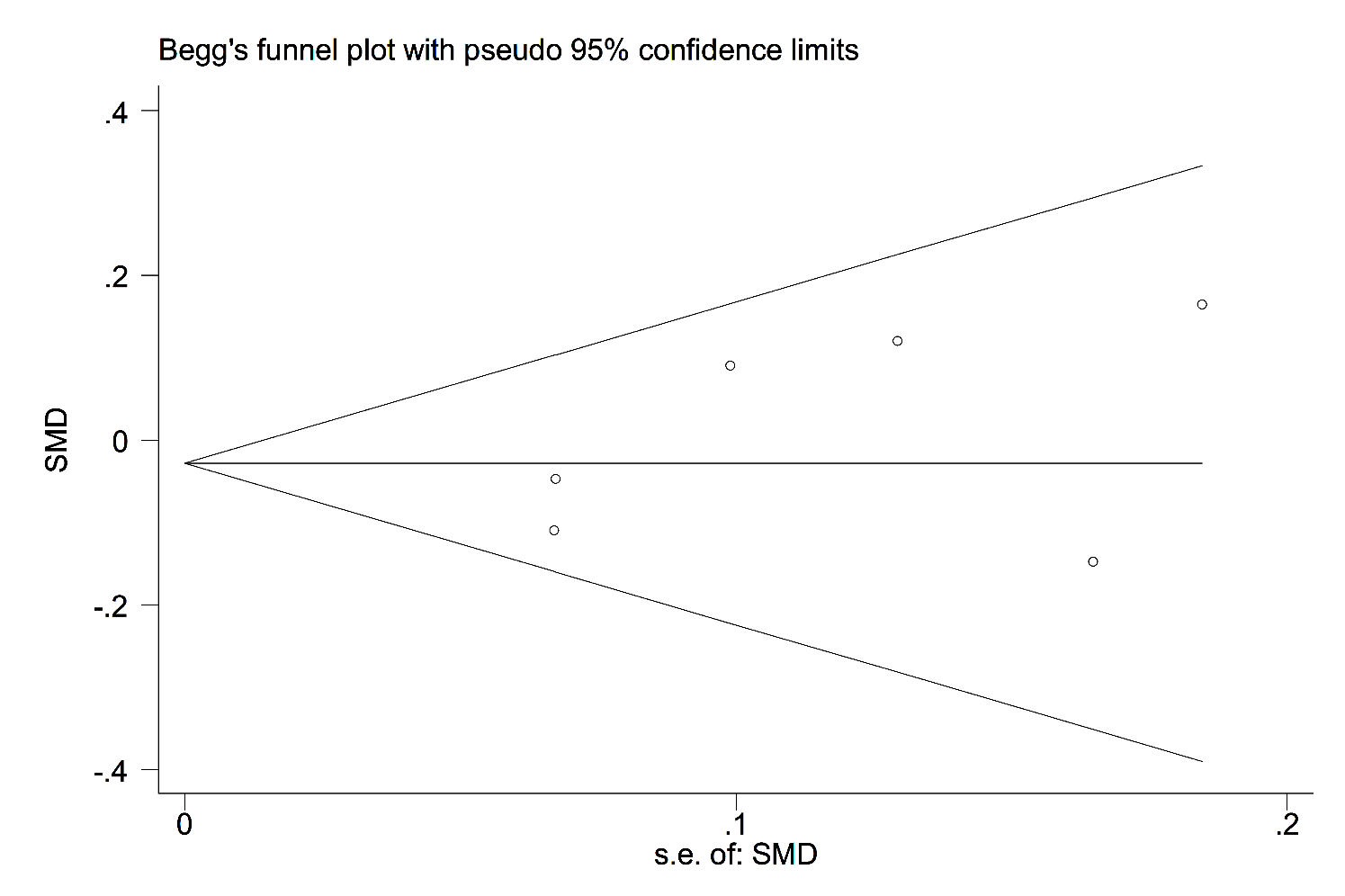
**SFig 18.** Begg’s funnel plot of the association analysis between *miRNA-499a* rs3746444 polymorphism and plasma TC levels.


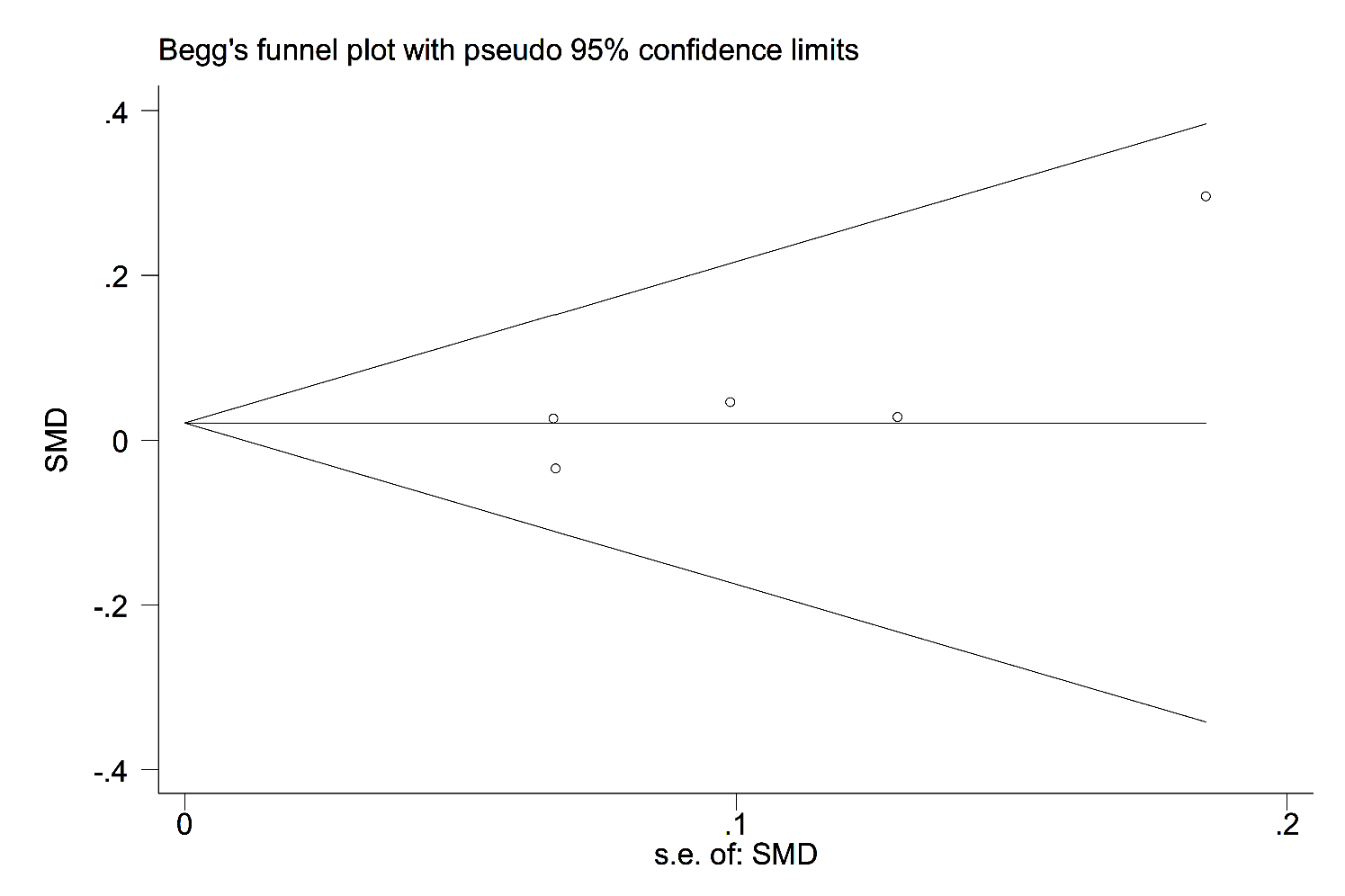
**SFig 19.** Begg’s funnel plot of the association analysis between *miRNA-499a* rs3746444 polymorphism and plasma LDL-C levels.


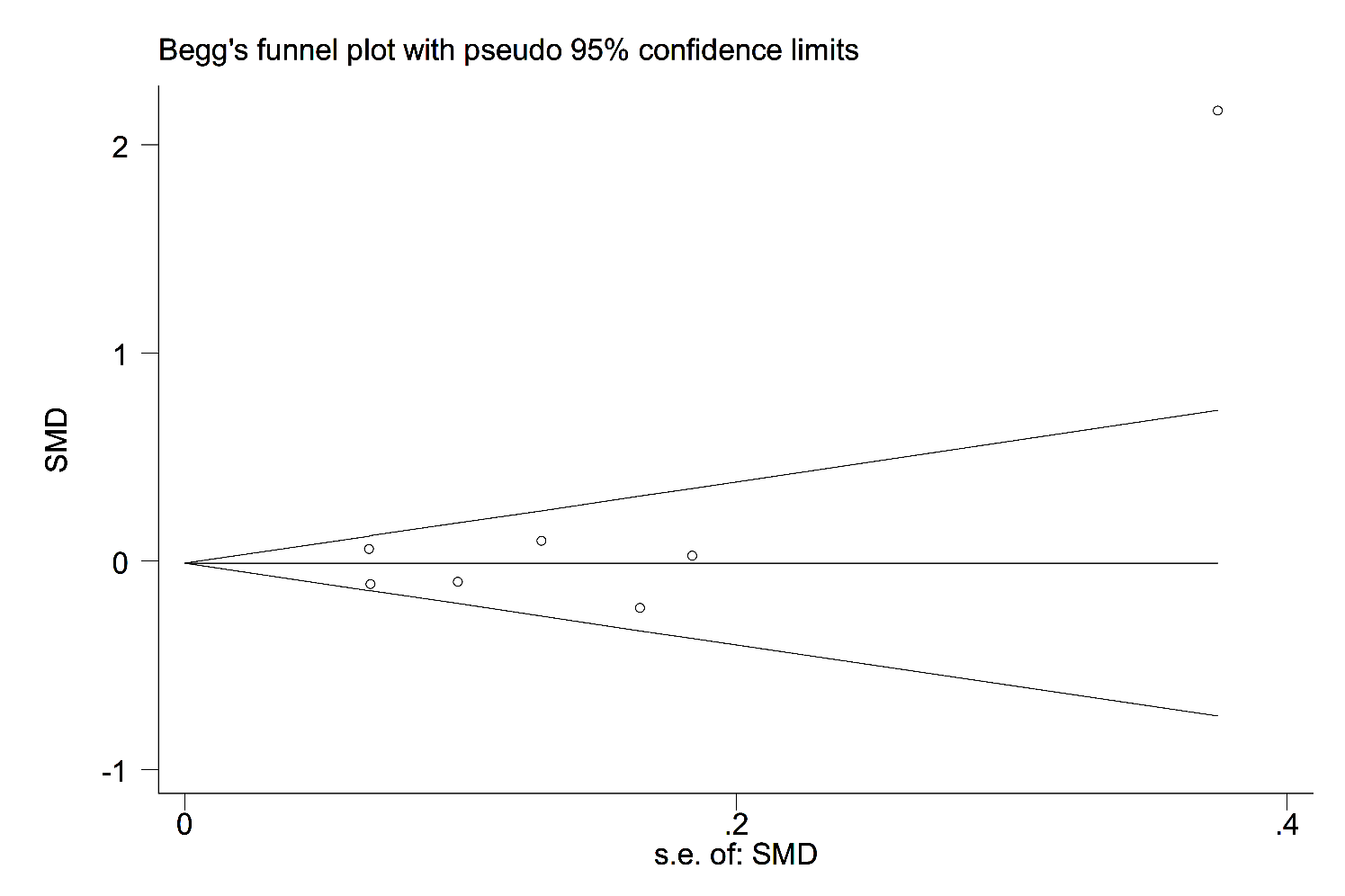
**SFig 20.** Begg’s funnel plot of the association analysis between *miRNA-499a* rs3746444 polymorphism and plasma HDL-C level.

**Supplemental References**

R1. Li Q, Chen L, Chen D, Wu X, Chen M. Influence of microRNA-related polymorphisms on clinical outcomes in coronary artery disease. Am J Transl Res. 2015;7(2):393-400.

R2. Mehanna ET, Ghattas MH, Mesbah NM, Saleh SM, Abo-Elmatty DM. Association of MicroRNA-146a rs2910164 Gene Polymorphism with Metabolic Syndrome. Folia Biol (Praha). 2015;61(1):43-8.

R3. Alipoor B, Meshkani R, Ghaedi H, Sharifi Z, Panahi G, Golmohammadi T. Association of miR-146a rs2910164 and miR-149 rs2292832 Variants with Susceptibility to Type 2 Diabetes. Clin Lab. 2016;62(8):1553-1561.

R4. Chen LB, Zheng HK, Zhang L, An Z, Wang XP, Shan RT, Zhang WQ. A single nucleotide polymorphism located in microRNA-499a causes loss of function resulting in increased expression of osbpl1a and reduced serum HDL level. Oncol Rep. 2017;38(6):3515-3521.

R5. Luo HC, Luo QS, Wang CF, Lei M, Li BL, Wei YS. Association of miR-146a, miR-149, miR-196a2, miR-499 gene polymorphisms with ischemic stroke in a Chinese people. Oncotarget. 2017;8(46):81295-81304.

R6. Chen CM, Dong SH, Li HM, Chen TT, Liu HD, Xiong W. The correlation of microRNA -146a gene polymorphism and acute coronary syndrome. Journal of Jilin Medicine. 2018; 39: 2045-2047.

R7. Ciccacci C, Latini A, Greco C, Politi C, D'Amato C, Lauro D, Novelli G, Borgiani P, Spallone V. Association between a MIR499A polymorphism and diabetic neuropathy in type 2 diabetes. J Diabetes Complications. 2018;32:11-17.

R8. Abo-Elmatty DM, Mehanna ET. MIR146A rs2910164 (G/C) Polymorphism is Associated with Incidence of Preeclampsia in Gestational Diabetes Patients. Biochem Genet. 2019;57(2):222-233.

R9. Qiu XY, Lu ST, Fan MK, Geng HH, Han ZY, Gao SP, Pan HY, Huang R, Pan M. Effects of Polymorphisms in Pre-miRNA on Inflammatory Markers in Atrial Fibrillation in Han Chinese. Clin Lab. 2019;65(7).

R10. Qiu H, Chen Z, Lv L, Tang W, Hu R. Associations Between microRNA Polymorphisms and Development of Coronary Artery Disease: A Case-Control Study. DNA Cell Biol. 2020;39(1):25-36.
